# Epistatic control of intrinsic resistance by virulence genes in *Listeria*

**DOI:** 10.1371/journal.pgen.1007525

**Published:** 2018-09-04

**Authors:** Mariela Scortti, Lei Han, Sonsiray Alvarez, Alexandre Leclercq, Alexandra Moura, Marc Lecuit, Jose Vazquez-Boland

**Affiliations:** 1 Microbial Pathogenesis Group, Division of Infection Medicine, Edinburgh Medical School (Biomedical Sciences), University of Edinburgh, Little France campus, Edinburgh, United Kingdom; 2 Division of Infection & Immunity, The Roslin Institute, University of Edinburgh, Easter Bush campus, Edinburgh, United Kingdom; 3 Institut Pasteur, Biology of Infection Unit, INSERM U111 and National Reference Centre / WHO Collaborating Centre for *Listeria*, Paris, France; 4 Paris Descartes University, Division of Infectious Diseases and Tropical Medicine, Necker-Enfants Malades University Hospital, Paris, France; Uppsala University, SWEDEN

## Abstract

Elucidating the relationships between antimicrobial resistance and virulence is key to understanding the evolution and population dynamics of resistant pathogens. Here, we show that the susceptibility of the gram-positive bacterium *Listeria monocytogenes* to the antibiotic fosfomycin is a complex trait involving interactions between resistance and virulence genes and the environment. We found that a FosX enzyme encoded in the listerial core genome confers intrinsic fosfomycin resistance to both pathogenic and non-pathogenic *Listeria* spp. However, in the genomic context of the pathogenic *L*. *monocytogenes*, FosX-mediated resistance is epistatically suppressed by two members of the PrfA virulence regulon, *hpt* and *prfA*, which upon activation by host signals induce increased fosfomycin influx into the bacterial cell. Consequently, in infection conditions, most *L*. *monocytogenes* isolates become susceptible to fosfomycin despite possessing a gene that confers high-level resistance to the drug. Our study establishes the molecular basis of an epistatic interaction between virulence and resistance genes controlling bacterial susceptibility to an antibiotic. The reported findings provide the rationale for the introduction of fosfomycin in the treatment of *Listeria* infections even though these bacteria are intrinsically resistant to the antibiotic *in vitro*.

## Author summary

Epistasis, or interactions between genes, is the phenomenon where the phenotypic effect of a locus is altered or masked by other loci in a given genomic context. Working with *Listeria* bacteria, we show that the effect of an intrinsic resistance determinant that protects these organisms against fosfomycin, a natural, microbial-derived antibiotic, is epistatically cancelled by virulence determinants present in the pathogenic species, *L*. *monocytogenes*. Since these virulence determinants are specifically activated within the host, the epistatic effect only manifests during infection, not when the bacteria are living saprophytically. Our study dissects the underlying mechanism, substantiating at the molecular level that virulence and resistance can be closely intertwined via gene-gene epistatic phenomena, with strong effects on the antimicrobial susceptibility phenotype. The findings are significant because any functional interaction between resistance and virulence may inextricably link the evolution of these two key pathogen traits. Understanding in detail these interactions is essential for predicting the evolutionary dynamics of resistance among pathogenic microbes or the impact of antimicrobial policies on drug-resistant virulent strains. In addition to their fundamental interest, our study provides the science-based evidence needed for the use of fosfomycin to treat listeriosis, a severe foodborne infectious disease, despite the causative bacteria showing strong resistance to the antibiotic.

## Introduction

The facultative intracellular pathogen *Listeria monocytogenes* is the causative agent of listeriosis, a foodborne infection characterized by severe clinical manifestations including meningoencephalitis, bacteremia, miscarriage and neonatal sepsis or meningitis [1–3]. The pathogenesis of listeriosis relies on a group of virulence genes that are co-ordinately regulated by the PrfA transcriptional activator [4]. PrfA-regulated genes are selectively induced within host cells through a mechanism involving cofactor-mediated allosteric switching of PrfA between weakly active (“Off”) and strongly active (“On”) states [5, 6]. PrfA regulation is both essential for the activation of the listerial virulence program within the host and for preventing the costly production of unneeded virulence factors when *L*. *monocytogenes* is living as an environmental saprotroph [7, 8]. Listeriosis is the foodborne infection with the highest mortality in the Western hemisphere despite hospital-based therapy (20–50%) [2]. This is partly attributable to the intracellular lifestyle of *L*. *monocytogenes* and the location of lesions, e.g. the brain, which render these bacteria relatively inaccessible to drugs thereby limiting the therapeutic choices [9, 10]. Cell-permeant antimicrobials able to penetrate the blood-brain barrier (BBB) and other listerial infection sites at bactericidal concentrations may therefore significantly aid in the treatment of listeriosis.

Previous work from our laboratory identified fosfomycin (disodium salt for parenteral use) [11–13] as one such potentially useful anti-listerial drugs. Fosfomycin [(1R,2S)-epoxypropylphosphonic acid] is a low-molecular-weight bactericidal molecule discovered in 1969 in *Streptomyces fradiae* [14] that inhibits peptidoglycan biosynthesis through covalent inactivation of UDP-*N-*acetylglucosamine-3-enolpyruvyl transferase (MurA) [11]. Although known to be resistant to fosfomycin by standard *in vitro* testing [9, 15, 16], we found that *L*. *monocytogenes* was actually susceptible to this antibiotic in infected cells and *in vivo* in mice [17]. The efficacy of fosfomycin against intracellular *L*. *monocytogenes* was independently confirmed by others [18]. The basis of this *in vitro*-*in vivo* paradox is the PrfA-regulated expression of the listerial sugar phosphate permease Hpt, a homolog of the enterobacterial hexose phosphate transporter UhpT that also transports fosfomycin [17]. Hpt is a virulence factor that promotes rapid replication in the cytosol by allowing bacterial utilization of host-cell hexose phosphates as a carbon source [19]. However, Hpt remains unexpressed outside the host due to PrfA On-Off switching [17, 20], preventing Hpt-mediated fosfomycin import into the listerial cell [17]. Importantly, we also showed that *L*. *monocytogenes* spontaneous fosfomycin resistance was mostly due to mutations in the *prfA* (56%) or *hpt* (41%) genes [17]. Since *prfA* is essential for pathogenesis [6, 21] and *hpt* is required for full *in vivo* virulence [19], *L*. *monocytogenes* fosfomycin resistant mutants were consequently found to be counterselected in infected macrophages [17].

Despite the above evidence, a potential obstacle for the clinical use of fosfomycin in the treatment of listeriosis is the reported presence in *L*. *monocytogenes* of a fosfomycin hydrolyzing enzyme, FosX [22]. The *fosX* gene was originally discovered in the soil bacteria *Mesorhizobium loti* and *Desulfitobacterium hafniense* and in the *L*. *monocytogenes* reference genome strain EGDe (*lmo1702*) by *in silico* mining for homologs of the fosfomycin resistance proteins FosA and FosB [23]. However, the actual distribution of *fosX*, and whether this gene actually confers fosfomycin resistance in *L*. *monocytogenes* had not been established.

In this study, we show that *fosX* is a core trait of the *Listeria* genus that confers high levels of resistance to fosfomycin unless epistatically controlled, in the pathogenic species *L*. *monocytogenes*, by members of the *in vivo*-activated PrfA virulence regulon. Our work demonstrates that epistatic interactions between virulence and resistance genes can have dramatic effects on the antimicrobial susceptibility phenotype of bacterial pathogens.

## Results

### *fosX* is part of the *Listeria* core genome

**Fig 1:**
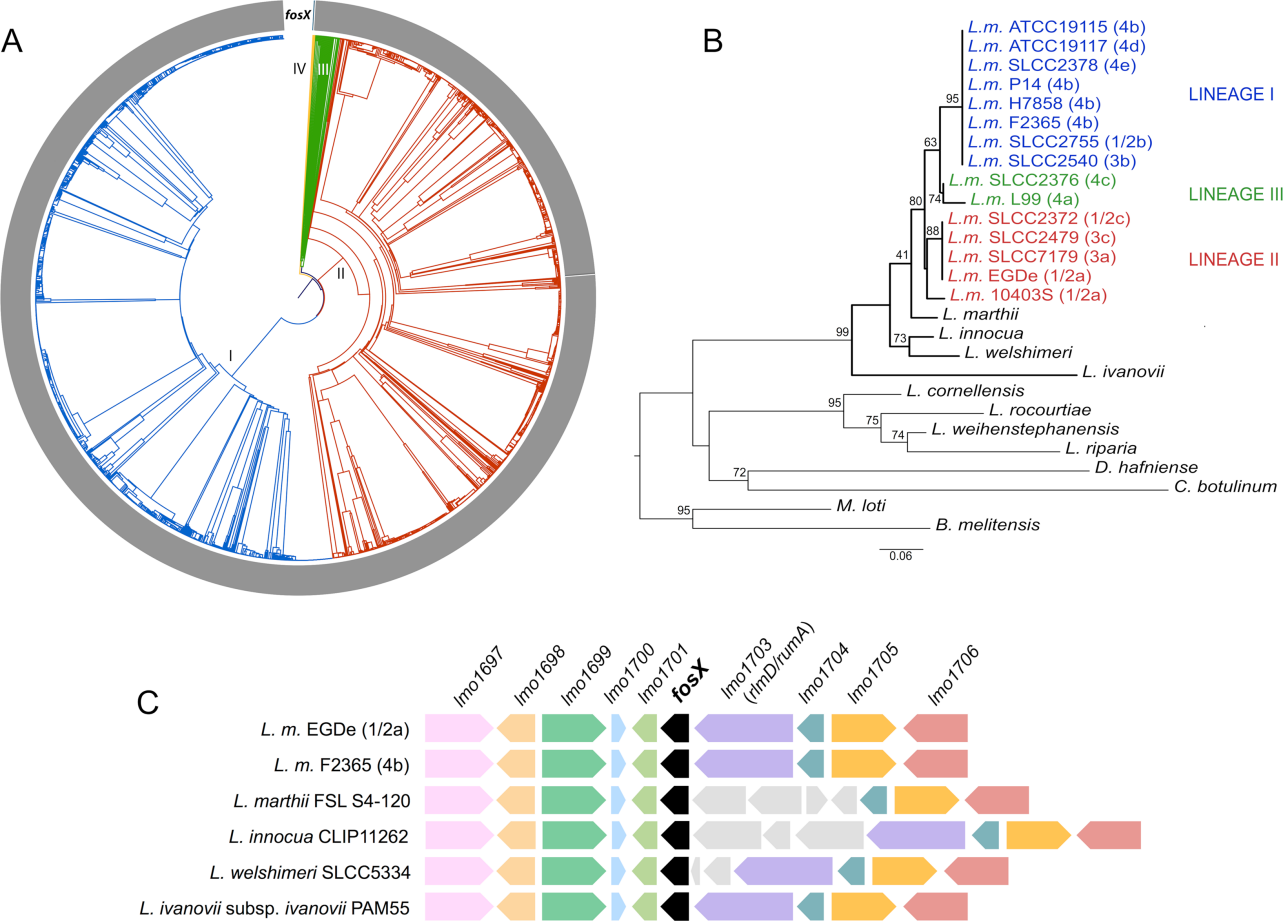
*fosX* evolved with the *Listeria* core genome. **(A)** Single linkage clustering of 1,696 *L*. *monocytogenes* isolates based on core-genome MLST profiles [24] showing the conservation of *foxX* in the species. Main *L*. *monocytogenes* lineages I to IV [24] are indicated in different colors. The presence of complete *fosX* coding regions is marked in gray in the outer ring (where gaps indicate strains with frameshift mutations leading to a truncated FosX protein; see text). **(B)** Neighbor-joining tree of FosX enzymes from *Listeria* spp. rooted with *Brucella melitensis* FosX (NCBI RefSeq WP_004687281.1). The topology of the tree mirrors the *Listeria* genus whole-genome phylogeny (which currently includes a number of *Listeria*-like groups) [25] and *L*. *monocytogenes* diversification into lineages (color coded as in panel A). The *Listeria sensu stricto* clade is indicated with thick lines. More distant *fosX* homologs (possibly paralogs) are present in the *Listeria sensu lato* “*Paenilisteria*” clade (represented by *L*. *cornellensis*, *L*. *rocourtiae*, *L*. *weihenstephanensis* and *L*. *riparia* in the tree; 63–65% amino acid sequence identity to *L*. *monocytogenes* FosX vs 73–91% for *Listeria sensu stricto* orthologs) in a different chromosomal location. See S1 Table for details. **(C)** Genomic organization around the *fosX* gene (*lmo1702*) in the *Listeria sensu stricto* clade [25]. Orthologs are in the same color, non-core genes are in gray. *fosX* is present in all members of the clade except *L*. *seeligeri*. *L*.*m*., *L*. *monocytogenes*. Coding sequence numbers according to *L*. *monocytogenes* EGDe nomenclature.

Analysis of a collection of genomic sequences from 1,696 *L*. *monocytogenes* isolates from 13 countries, representing the four lineages of the species, 164 sublineages and 1,013 core-genome MLST subtypes [24], showed that the *fosX* gene is universally conserved in *L*. *monocytogenes* (Fig 1A). *L*. *monocytogenes* strains encoded a 133-residue FosX protein between 92 and 100% identical to the product of the 402-bp *fosX* gene of strain EGDe [23] (S1 Table). No other putative fosfomycin resistance enzyme genes were identified in *L*. *monocytogenes*. FosX orthologs were also encoded in *Listeria innocua*, *Listeria welshimeri* (89% identity), *Listeria marthii* (91% identity) and *Listeria ivanovii* (73% identity) (S1 Table). These species belong together with *L*. *monocytogenes* to one of the main phylogenetic subdivisions of the genus, clade (i) or *Listeria* “*sensu stricto*” [25]. With the exception of *Listeria seeligeri*, in which the gene appears to have been lost, *fosX* was present at the same chromosomal location in all members of the *Listeria sensu stricto* clade (Fig 1C). This, and the fact that a phylogenetic tree based on the FosX protein sequence closely mirrored the genus’ phylogenomic structure (Fig 1B), indicated that *fosX* is an ancient *Listeria* trait that evolved with the core genome of these bacteria.

### *fosX* confers intrinsic resistance to fosfomycin in *Listeria*

**Fig 2:**
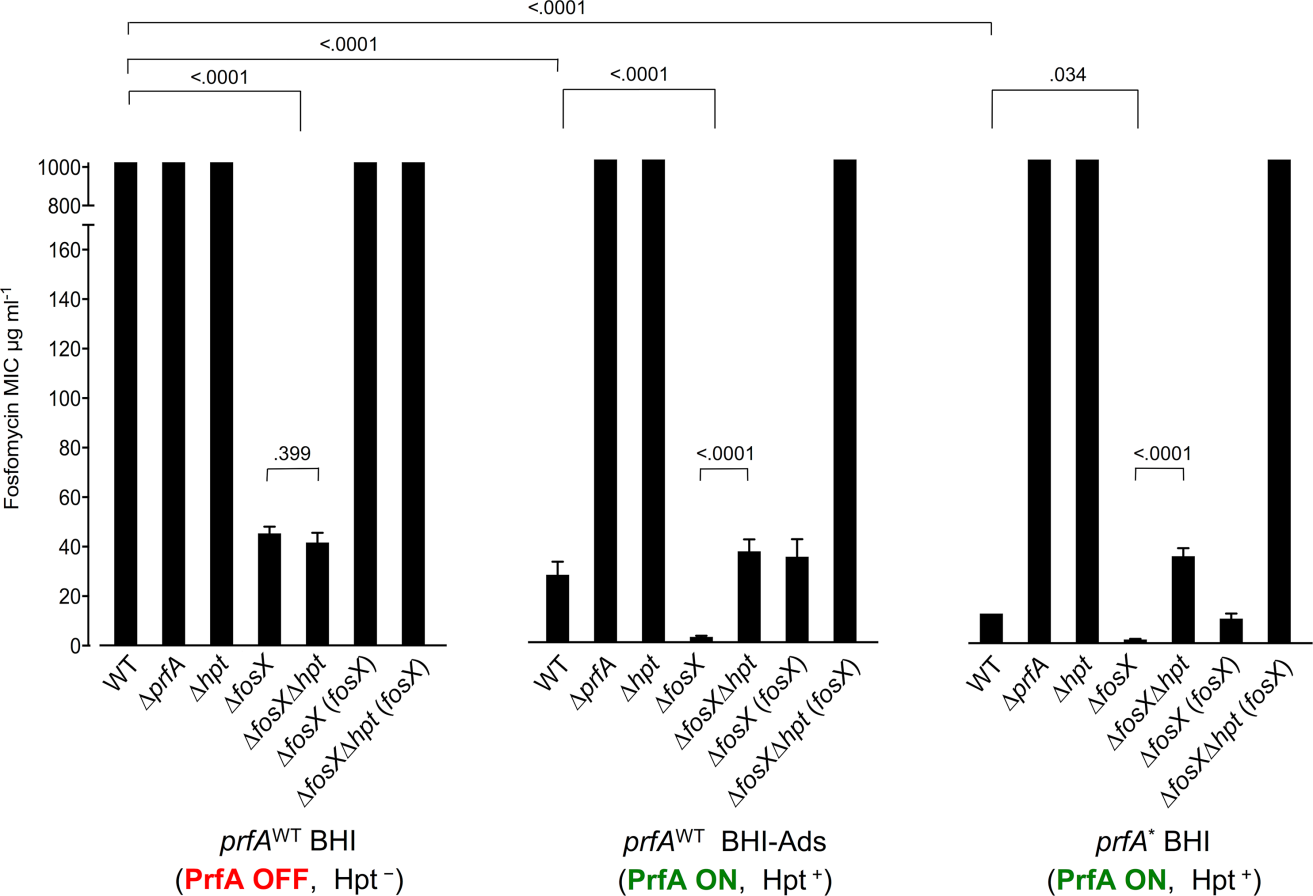
Genetic analysis of the role of *fosX*, *prfA* and *hpt* in *L*. *monocytogenes* fosfomycin phenotype. MIC, minimal inhibitory concentration. Mean ± SD from at least three independent experiments. Relevant *P* values are indicated (one-way ANOVA with uncorrected Fisher’s post-hoc multiple comparisons). Left panel: wild-type *prfA* allele (WT) in BHI medium (virulence-resting conditions i.e. PrfA system “Off”, Hpt not expressed). Middle panel: WT in charcoal-supplemented BHI (BHI-Ads; virulence-activating conditions i.e. PrfA system “On”, Hpt expressed). Right panel: constitutively activated prfA* allele in BHI (*in vivo*-like virulence-activating conditions i.e. PrfA locked “On”, Hpt expressed). See S2 Table for bacterial constructs. Note: (i) in *in vitro* (outside host) conditions (left panel), deletion of *fosX* renders *L*. *monocytogenes* susceptible to fosfomycin (drop in MIC from ≥1,024 to 45.3 μg/ml; susceptibility breakpoint = 64 μg/ml [18]); (ii) in infection-like conditions (middle and right panels), the activation of the PrfA system and ensuing increased influx of fosfomycin via the PrfA-regulated organophosphate transporter Hpt overrides *fosX*-mediated resistance and *L*. *monocytogenes* becomes susceptible to fosfomycin (MICs between 12.0 and 27.3 μg/ml).

**Fig 3:**
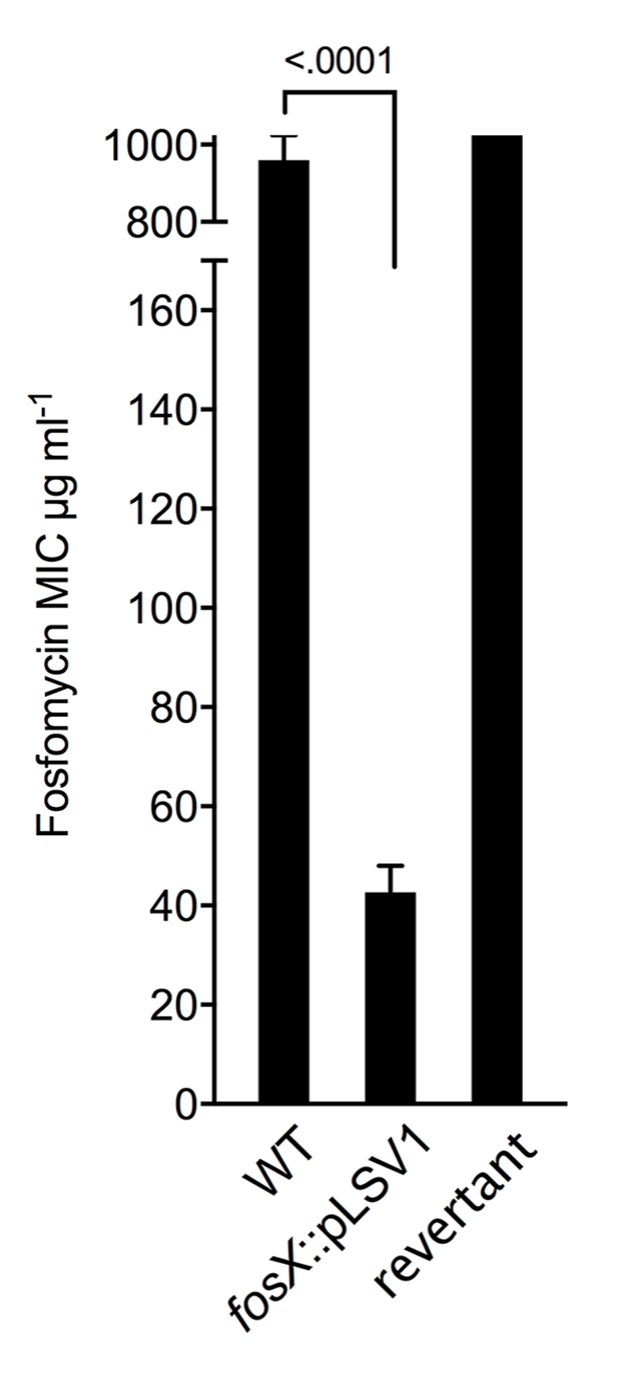
*fosX* confers intrinsic fosfomycin resistance to non-pathogenic *Listeria*. *L*. *innocua* strain CLIP 11262 was used as representative obligate saprotrophic *Listeria* sp. Data for wild type, *fosX* knock-out mutant by pLSV1 plasmid insertion, and revertant thereof in BHI. Identical results were obtained in BHI-Ads. Mean ± SD from three experiments. Relevant *P* values are indicated (One-way ANOVA with Dunnett’s multiple comparisons).

To investigate the role of *fosX* in the *L*. *monocytogenes* fosfomycin phenotype, we constructed an in-frame deletion mutant in *L*. *monocytogenes* P14, a serovar 4b human clinical isolate (S2 Table). The *fosX* null mutation caused a strong reduction in the fosfomycin minimum inhibitory concentration (MIC) in brain-heart infusion (BHI), from ≥1024 of the parental P14 to 45.3 μg/ml (*P* < 0.0001) (Fig 2, left panel). Knocking out *fosX* in the representative non-pathogenic species *L*. *innocua*, which like *L*. *monocytogenes* shows intrinsic fosfomycin resistance, had the same effect (Fig 3). Complementation of P14Δ*fosX* with a single copy of *fosX* expressed from its natural promoter (S1 Fig) restored wild-type MIC levels (Fig 2, left panel), demonstrating that FosX confers strong resistance to fosfomycin on *Listeria*.

**Fig 4:**
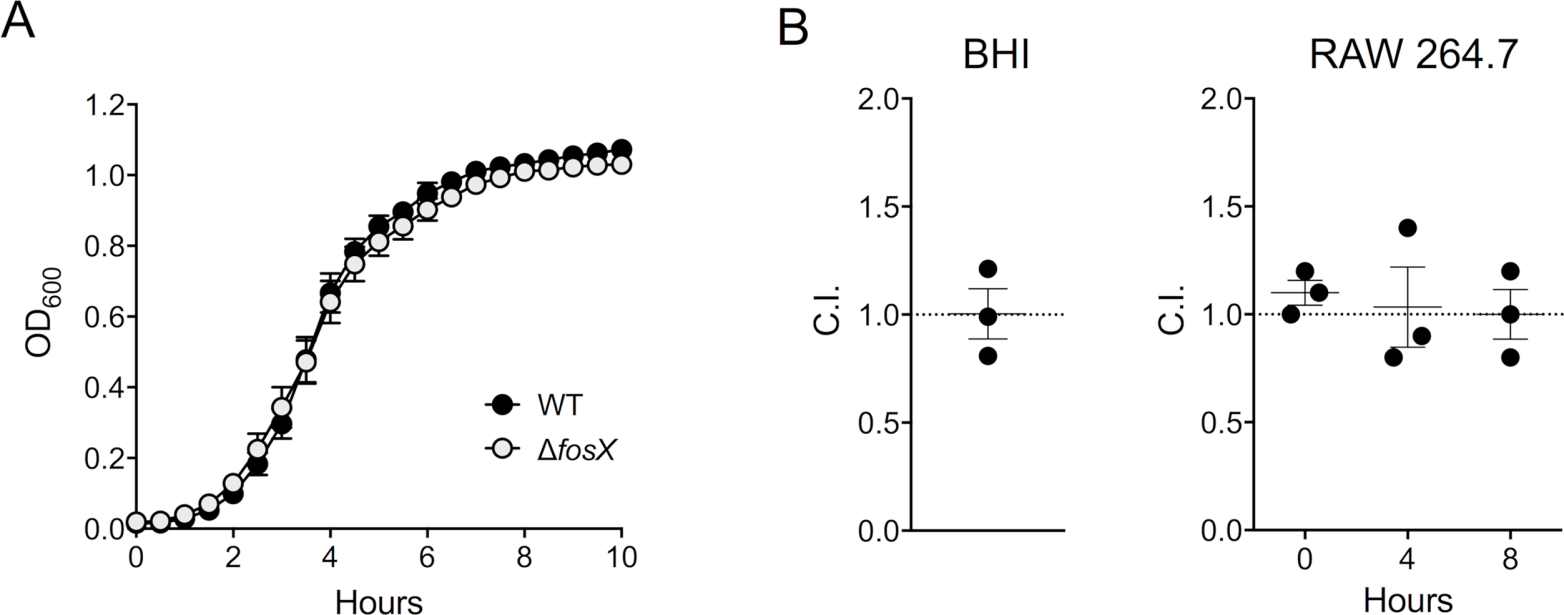
*fosX* is fitness neutral. **(A)** Growth curves of *L*. *monocytogenes* P14 (WT) and isogenic Δ*fosX* mutant in BHI. Mean ± SEM of five duplicate experiments. **(B)** Competition experiments between P14 and Δ*fosX* in BHI (left panel) and RAW 264.7 mouse macrophages (right panel). BHI broth or RAW 264.7 cell monolayers were inoculated with a 1:1 mix of both bacteria and the competition indexes (C.I.) determined after incubation of 18 h at 37 °C or at the indicated intracellular infection time points. *t* = 0 corresponds to 1 h after infection (30 min incubation after addition of inoculum and centrifugation plus 30 min gentamicin treatment). Mean ± SEM of three duplicate experiments, a C.I. = 1 indicates equal competing ability. One-sample Student´s *t* test (hypothetical value 1, two tails) *P* values: BHI = 0.977; macrophages = 0.2254, 0.874 and ≥0.999 for *t* = 0, 4 and 8 h, respectively. Similar results as in BHI were obtained using less rich media (Luria-Bertani broth, C.I. = 0.92± 0.05, *P* = 0.331; chemically-defined IMM medium, C.I. = 1.037±0.071, *P* = 0.657).

The distribution and high degree of conservation of *fosX* among pathogenic and obligate saprotrophic *Listeria* spp. could be linked to the resistance phenotype, or might be related to other potential roles of the FosX enzyme in listerial physiology, as suggested for the components of the bacterial intrinsic resistomes [26–28]. For example, FosX from the soil bacterium *M*. *loti* is catalytically promiscuous, has a comparatively low capacity to hydrolyze fosfomycin, and is probably primarily involved in rhizobial metabolism, as indicated by the presence of the coding gene in a *phn* operon presumably involved in transport and utilization of phosphonate [22, 23]. We investigated possible homeostatic roles of *Listeria* FosX using competition experiments between wild-type and Δ*fosX L*. *monocytogenes* in broth medium and in infected macrophages in the absence of fosfomycin pressure. FosX was in both cases fitness neutral (Fig 4), indicating that it has no significant housekeeping function, at least in our experimental conditions.

Interestingly, *L*. *monocytogenes* transcription start site mapping data [29] indicate that *fosX* (*lmo1702*) is co-expressed with the upstream gene *lmo1703* encoding a putative TrmA superfamily SAM-dependent RNA methyltransferase similar to RlmD/RumA [30] (S1 Fig). This gene arrangement is unique to *Listeria*; in fact, *fosX* genes are found in completely different genetic environments in each of the bacterial taxa that carry this determinant (S2 Fig). Modification of specific rRNA nucleotides by methyltransferases plays a critical role in ribosomal function regulation and is also a well-known mechanism conferring resistance to ribosomal antibiotics [31–33]. Specifically, mutagenesis studies of the RmlD/RumA uridine 1939 target in the *Escherichia coli* 23S ribosomal subunit demonstrated altered susceptibility patterns to antibiotics that affect protein synthesis [34]. Fosfomycin is produced by several species of the ubiquitous environmental microbes *Streptomyces* and *Pseudomonas* [11, 14], which *Listeria* spp. are likely to encounter in the natural habitat. It is tempting to speculate that *fosX* and the conserved adjacent *rmlD/rumA*-like homolog found in *Listeria* spp. form a “chromosomal resistance” island conferring simultaneous protection against microbially derived fosfomycin and ribosome-targeting secondary metabolites. It cannot be excluded, however, that the enzymatic activity of FosX may play a more general role in bacterial physiology by mediating epoxide ring hydrolysis (23) in some catabolic processes relevant to *Listeria*.

### Evidence for OPA permease-independent fosfomycin import

**Fig 5:**
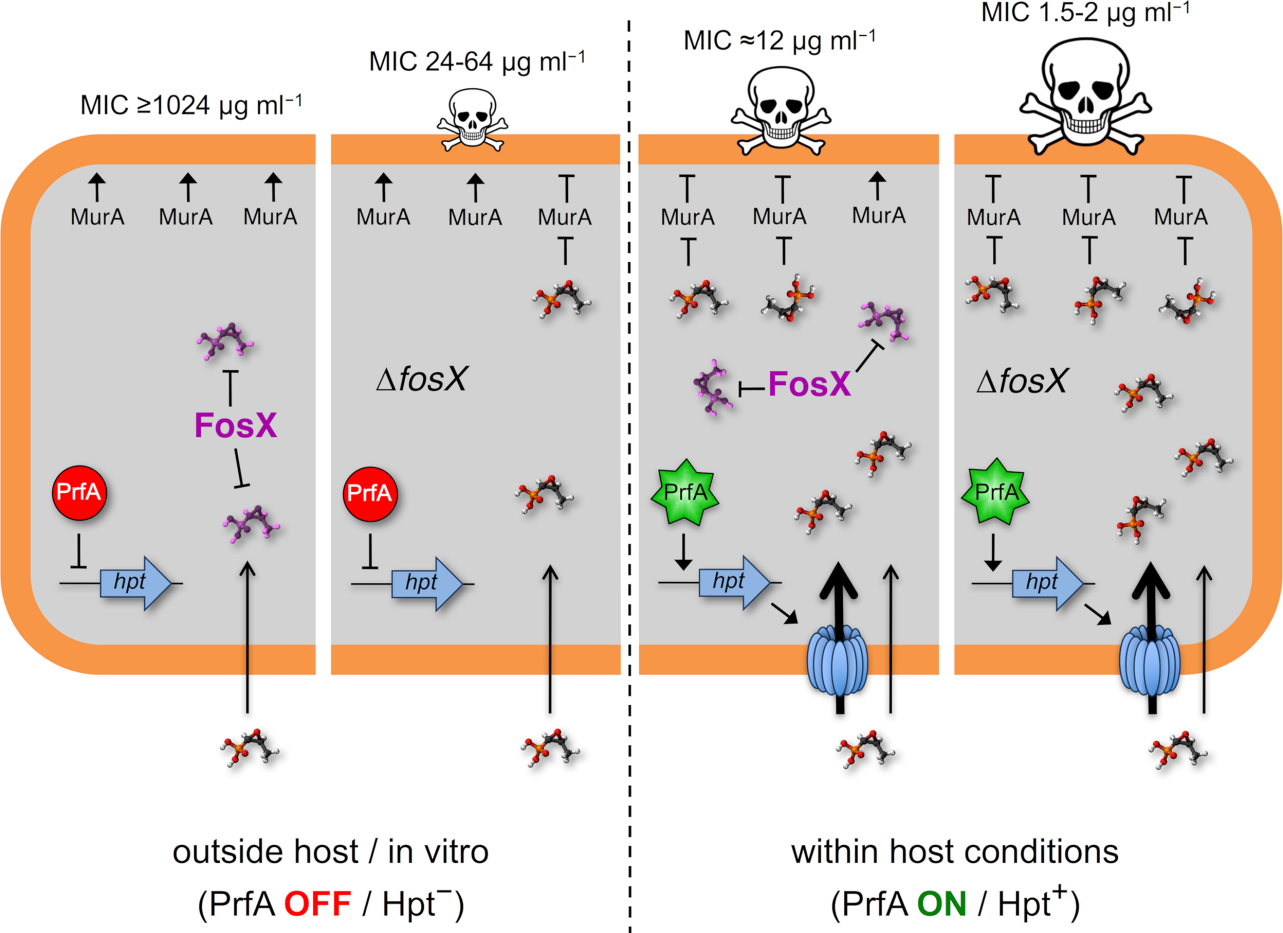
Model of virulence-resistance gene epistatic interaction determining *L*. *monocytogenes* fosfomycin phenotype. In saprophytic (*in vitro*) conditions (left section of figure), the FosX enzyme inactivates the (relatively low) concentrations of fosfomycin that enter the bacterial cell via an uncharacterized transport mechanism (indicated with an inward arrow; see text for details). This fosfomycin uptake mechanism operates in both obligate saprotrophic and pathogenic *Listeria* spp. However, in *L*. *monocytogenes*, two of its virulence determinants, *prfA* encoding the central virulence regulator PrfA [4], and the PrfA-regulated *hpt* gene encoding the sugar phosphate permease Hpt [19] (which also transports fosfomycin) [17], suppress the effect of FosX. This occurs during infection, where PrfA is activated upon sensing of host signals [5, 6, 41] and PrfA-promoted expression of Hpt leads to increased fosfomycin influx. In these conditions, FosX is overwhelmed and a fraction of incoming fosfomycin reaches its MurA target at inhibitory concentrations (right section of figure). Fosfomycin is shown in molecular structure representation. MurA catalyzes the first committed step in peptidoglycan biosynthesis (ligation of phosphoenolpyruvate to the 3’-hydroxy group of UDP-*N*-acetylglucosamine) [11, 36].

The fosfomycin susceptibility of P14Δ*fosX* in BHI raises the question of how fosfomycin might enter the listerial cell at inhibitory concentrations in conditions where PrfA is “Off” and Hpt is completely downregulated [17]. A double Δ*fosX*Δ*hpt* mutant had the same MIC as the *fosX* mutant (*P* = 0.399) (Fig 2, left panel), excluding leaky expression of the Hpt transporter as the cause. Although a very small molecule (138 Da), fosfomycin is hydrophilic and unlikely to permeate into the bacterial cell unless through facilitated diffusion via a carrier protein(s). The only known bacterial fosfomycin transporters are two types of organophosphate:inorganic phosphate antiporters (OPA) [35], exemplified by the hexose phosphate transporter UhpT (and listerial homologue Hpt) and the GlpT glycerol-3-phosphate permease [11, 36]. Genome searches confirmed that Hpt is the only OPA permease in *Listeria* spp. This indicates that the susceptibility of the *L*. *monocytogenes* Δ*fosX*Δ*hpt* mutant (and the *L*. *innocua fosX* mutant) must depend on another, as yet unknown fosfomycin uptake pathway (Fig 5). We suggest that the selective pressure imposed by this uncharacterized transport mechanism is a major driver underlying *fosX* acquisition and maintenance in *Listeria*.

### Epistasis of the virulence genes *prfA* and *hpt* over *fosX*

We tested the effect of *fosX* when Hpt-mediated fosfomycin transport is active using two “infection-mimicking” *in vitro* conditions: (i) supplementation of BHI medium with an adsorbent (activated charcoal or Amberlite XAD-4), which causes the partial activation of the PrfA regulation system by an as yet not fully understood mechanism [37]; and (ii) use of a constitutively activated prfA* allele, where a single amino acid substitution (e.g. PrfA*^G145S^) locks PrfA in “On” state, causing constitutive activation of the PrfA-regulated virulence genes [38]. A strong reduction in the fosfomycin MIC is typically observed with each of these two PrfA-activating strategies [17] (from ≥1024 to 27.3±5.3 and 12±0 μg/ml, respectively; *P* < 0.0001) (Fig 2, middle and right panels). In these conditions, the *fosX* mutation lowered further the fosfomycin MIC to virtually complete susceptibility (2.2±0.4 and 1.5±0.4 μg/ml, respectively) (*P* = 0.01). Complementation of the Δ*fosX* mutant restored the MIC to parental levels (Fig 2, middle and right panels). As expected [17], deletion of *hpt* or its transcriptional activator gene *prfA* rendered *L*. *monocytogenes* resistant to fosfomycin (MIC >1,024 μg/ml) (Fig 2, middle and right panels) but had no effect on bacteria with a wild-type *prfA* allele in BHI (where PrfA is “Off” and *hpt* is not expressed) (Fig 2, left panel). Ablation of Hpt function in the FosX^−^background under conditions of PrfA activation raised the MIC, from 2.2±0.4 to 36.8±7.0 μg/ml (charcoal-supplemented BHI [BHI-Ads]) or 1.5±0.4 to 35.2±6.4 μg/ml (prfA* allele) (P <0.001). These higher MIC values were similar to those for Δ*fos*X (or the double mutant Δ*fos*XΔ*hpt*) in BHI (45.3±2.7 μg ml/ml and 41.6±3.9, respectively) (Fig 2).

Overall, the above findings are consistent with a scenario where FosX: (i) can successfully inactivate the amounts of fosfomycin that enter the bacterial cell via the uncharacterized “constitutive” transport system, conferring complete resistance in *in vitro* (saprophytic) conditions; but (ii) is unable to process an increased influx of fosfomycin molecules via the Hpt transporter in PrfA-activating (infection) conditions (Fig 5). In other words, our data show that the intrinsic resistance conferred by the *fosX* gene is masked, or epistatically supressed, by the joint effect of the virulence genes *prfA* and *hpt* on fosfomycin transport.

### Epistatic effect during infection

**Fig 6:**
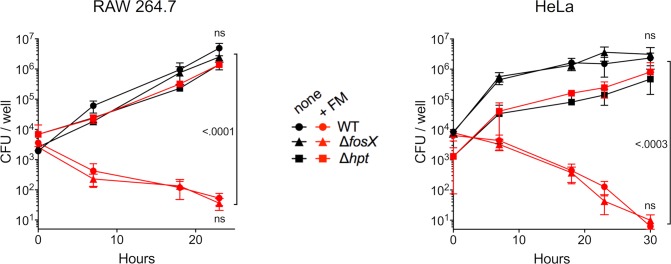
Fosfomycin susceptibility in infected cells. Intracellular survival of wild-type P14 (WT) and isogenic Δ*fosX* mutant demonstrating the lack of effect of FosX on *L*. *monocytogenes* susceptibility during infection. Experiments were conducted in RAW 264.7 macrophages and HeLa epithelial cells incubated with and without 180 μg/ml fosfomycin. A Δ*hpt* mutant where PrfA-dependent fosfomycin uptake is disabled was included as a control. Mean ± SEM of at least three duplicate experiments. Relevant *P* values are indicated; ns, not significant (one-way ANOVA and Šídák tests of data at final time-point).

We next assessed the extent to which the epistatic effect that cancels *fosX*-mediated resistance manifests during infection, where *prfA/hpt* are naturally induced [5, 6, 39–41]. To this end, the intracellular susceptibilities of wild-type *L*. *monocytogenes* P14 and isogenic Δ*fosX* derivative (and Δ*hpt* mutant as a control) were compared in survival/proliferation assays in infected RAW 264.7 macrophages in the presence and absence of fosfomycin. Cell cultures were incubated with 5× the physiological concentration of D-glucose as in these conditions listerial intracellular growth is independent of Hpt-mediated hexose phosphate uptake [17]. As shown in Fig 6, both wild-type and Δ*fosX L*. *monocytogenes* were equally susceptible to fosfomycin during intracellular infection (*P* = 0.996). In contrast, fosfomycin had in these conditions no effect on the Δ*hpt* mutant with disabled Hpt transport. Identical results were obtained using the human epithelial cell line HeLa (Fig 6). These data confirmed that *L*. *monocytogenes* is fully susceptible to fosfomycin in infection conditions, specifically as a consequence of the epistatic supression of *fosX*-mediated resistance by the PrfA-regulated (*in vivo*-activated) *hpt* gene.

### PrfA activation does not affect *fosX* expression

**Fig 7:**
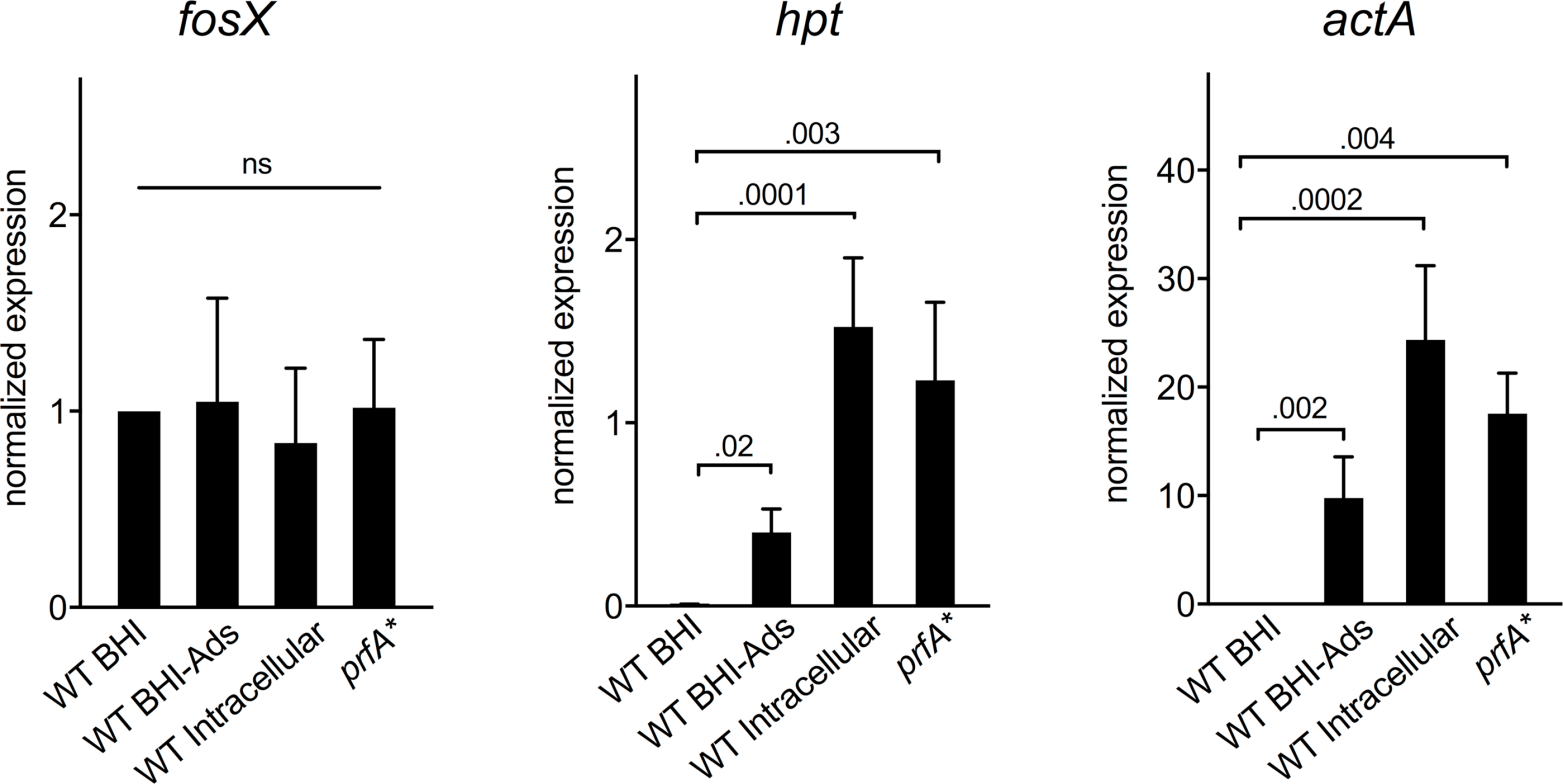
Constitutive expression of *fosX*. RT-QPCR transcription analysis of *fosX* and the PrfA-regulated *hpt* and (control) *actA* genes in *L*. *monocytogenes* P14 grown in BHI (PrfA “Off”) and under PrfA-inducing conditions (BHI-Ads, use of prfA*^G145S^ allele in BHI, intracellular infection in RAW 264.7 macrophages). BHI-Ads is Amberlite XAD-4-supplemented BHI. Mean ± SEM of at least three independent experiments in duplicate. Relevant *P* values are indicated (one-way ANOVA with uncorrected Fisher’s post-hoc multiple comparison).

While our data are consistent with the loss of *fosX*-mediated intrinsic resistance under PrfA induction (infection) conditions being primarily due to increased fosfomycin influx via Hpt (Fig 5), potential effects of PrfA (or the intracellular milieu) on *fosX* expression could also be a contributing factor. To explore this possibility, *fosX* transcription was analysed by RT-QPCR in BHI and in PrfA-activating conditions *in vitro* (adsorbent-treated medium, prfA* allele; see above) or during intracellular infection. All three PrfA-inducing conditions caused the expected transcriptional activation of the PrfA-regulated *hpt* and (control) *actA* genes [5, 42], with no significant changes in *fosX* expression (*P* = 0.615) (Fig 7). These data excluded a potential involvement of reduced expression of *fosX* in the susceptibility phenotype elicited by PrfA activation.

### Fosfomycin susceptibility of *L*. *monocytogenes* isolates under PrfA activation

**Fig 8:**
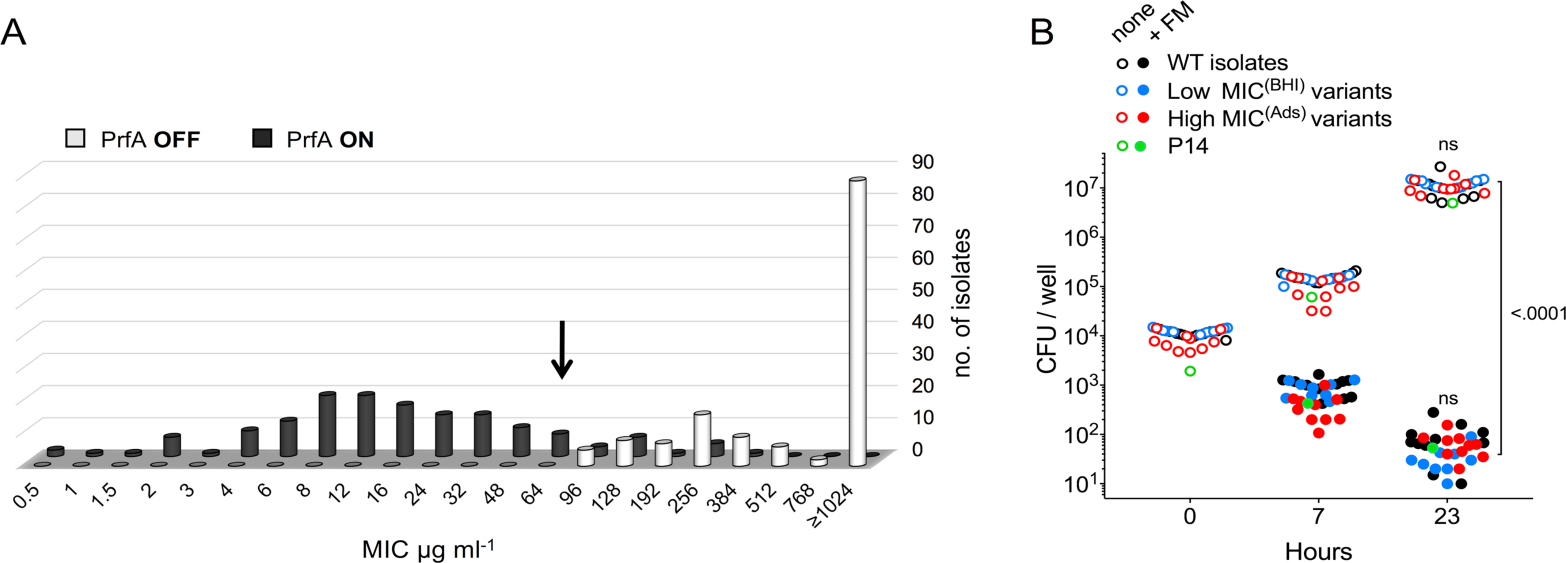
Fosfomycin susceptibility of *L*. *monocytogenes* isolates. **(A)** MICs of a panel of 142 wild-type isolates in BHI (PrfA “Off”, Hpt^–^) and charcoal-supplemented BHI (BHI-Ads; PrfA “On”, Hpt^+^). **(B)** Intracellular survival of *L*. *monocytogenes* isolates in RAW 264.7 macrophages in the presence and absence of fosfomycin (180 μg/ml). Three distinct sets of bacterial isolates with different fosfomycin phenotype were included: (i) “normal” adsorbent-activable fosfomycin susceptibility (WT, *n* = 11, strains listed in S3 Table), (ii) constitutively susceptible fosfomycin phenotype (“low MIC^(BHI)^ variants”, *n* = 9 including eight spontaneous *fosX* mutants, S3 Table), and (iii) isolates showing a relatively elevated MIC (96–384 μg/ml) in BHI-Ads (“high-MIC^(Ads)^ variants”, *n =* 10). Data for P14 are those from Fig 6, included for reference. Mean from at least two independent experiments. Relevant *P* values are indicated (two-way ANOVA with Dunnett´s multitple comparison tests); ns, not significant.

To establish whether the limited effect of *fosX* when the PrfA system is “On” and Hpt is expressed is a general feature of the *L*. *monocytogenes* species, the fosfomycin MIC of 142 wild-type isolates was tested in BHI and BHI-Ads. The MIC_50_ and MIC_90_ values shifted from ≥1,024 μg/ml in BHI to 16 and 64 μg/ml, respectively, in BHI-Ads (Fig 8A). Thus, despite *fosX*, in conditions of PrfA activation the fosfomycin MIC remained within the limits of susceptibility for the vast majority of the tested strains (90.33% with 64 μg/ml PK/PD breakpoint [18]; 80.64% with 32 μg/ml general fosfomycin breakpoint for gram-positive bacteria [43]). It must be noted that adsorbents only partially activate PrfA ([41] and Fig 7), and significantly lower fosfomycin MICs (median 3 μg/ml, range 2–16) are observed in *L*. monocytogenes prfA*^G145S^ bacteria with the PrfA system constitutively activated to *in vivo* (within-host)-like levels [17, 41] (see Fig 2, right panel).

To determine if the above findings can be extrapolated to infection conditions, the intracellular susceptibility of a selection of *L*. *monocytogenes* strains with “normal” (i.e. adsorbent-activable) fosfomycin phenotype was analysed in RAW 264.7 macrophages. The tested bacteria included eight wild-type human clinical isolates plus the well-characterized strains EGDe (serovar 1/2a), 10403 (serovar 1/2a) and CLIP 80459 (serovar 4b). In addition, we also tested a representation (*n* = 10) of the small proportion of isolates where the fosfomycin MICs remained relatively elevated (96–384 μg/ml) despite being BHI-Ads responsive, to determine if this correlated with differences in intracellular susceptibility. Fig 8B shows that all tested strains were equally susceptible to fosfomycin in infected macrophages (*P* = 0.632). These data confirmed that *L*. *monocytogenes* isolates are characteristically susceptible to fosfomycin during infection, even if the MIC remains above the 64-μg/ml breakpoint as long as they exhibit the capacity to respond to PrfA-activating conditions (as tested in BHI-Ads medium).

### *fosX* mutations in constitutively susceptible *L*. *monocytogenes* isolates

A percentage of *L*. *monocytogenes* clinical isolates exhibit constitutively low fosfomycin MICs under normal *in vitro* testing conditions [16] (about 2 to 4.5%; data from *L*. *monocytogenes* antibiotic susceptibility surveillance at Institut Pasteur’s National and WHO Collaborating Reference Centre for *Listeria* and ref. [18]). We examined nine human isolates carrying a *fosX* gene but presenting a fosfomycin MIC ≤64 μg/ml in PrfA-non-inducing conditions (normal BHI) to determine the underlying mechanism (S3 Table). All displayed a wild-type PrfA phenotype (see Materials and Methods), excluding possible spontaneous prfA* (*hpt*-activating) mutations as the cause for their constitutive *in vitro* fosfomycin susceptibility [17]. Consistent with the key role of the FosX enzyme in the intrinsic *in vitro* resistance of *L*. *monocytogenes* to fosfomycin, eight of the nine strains analyzed carried *fosX* mutations (S3 Table). The only isolate with wild-type FosX gave a “slow-positive” sugar-phosphate acidification test (S3 Table), pointing to an increased Hpt activity as the cause. However, no differences in *hpt* gene expression (S3 Fig) or in the DNA sequence of the *hpt* region that could explain the Hpt^(+)^ phenotype (S3 Table) were identified. Seven of the mutants had a premature stop codon at *fosX* triplet 128, leading to a truncated product where the lack of the six C-terminal residues most likely destabilizes FosX's catalytic site [22] (S4 Fig). The other *fosX* mutant carried a frameshift mutation at position 88 that introduced premature stop codons from position 89. Complementation analysis in P14Δ*fosX* confirmed that the *fosX*^128stop^ and *fosX*^88frameshift^ mutant alleles did not encode active FosX enzymes (BHI MICs: 48 and 32 μg/ml, respectively instead of ≥1,024 with wild-type *fosX*). As expected, similar to the P14Δ*fosX* (Fig 6), all the spontaneous *fosX* mutants showed complete susceptibility to fosfomycin in infected host cells (Fig 8B).

### Effect of *fosX* overexpression on fosfomycin susceptibility

**Fig 9:**
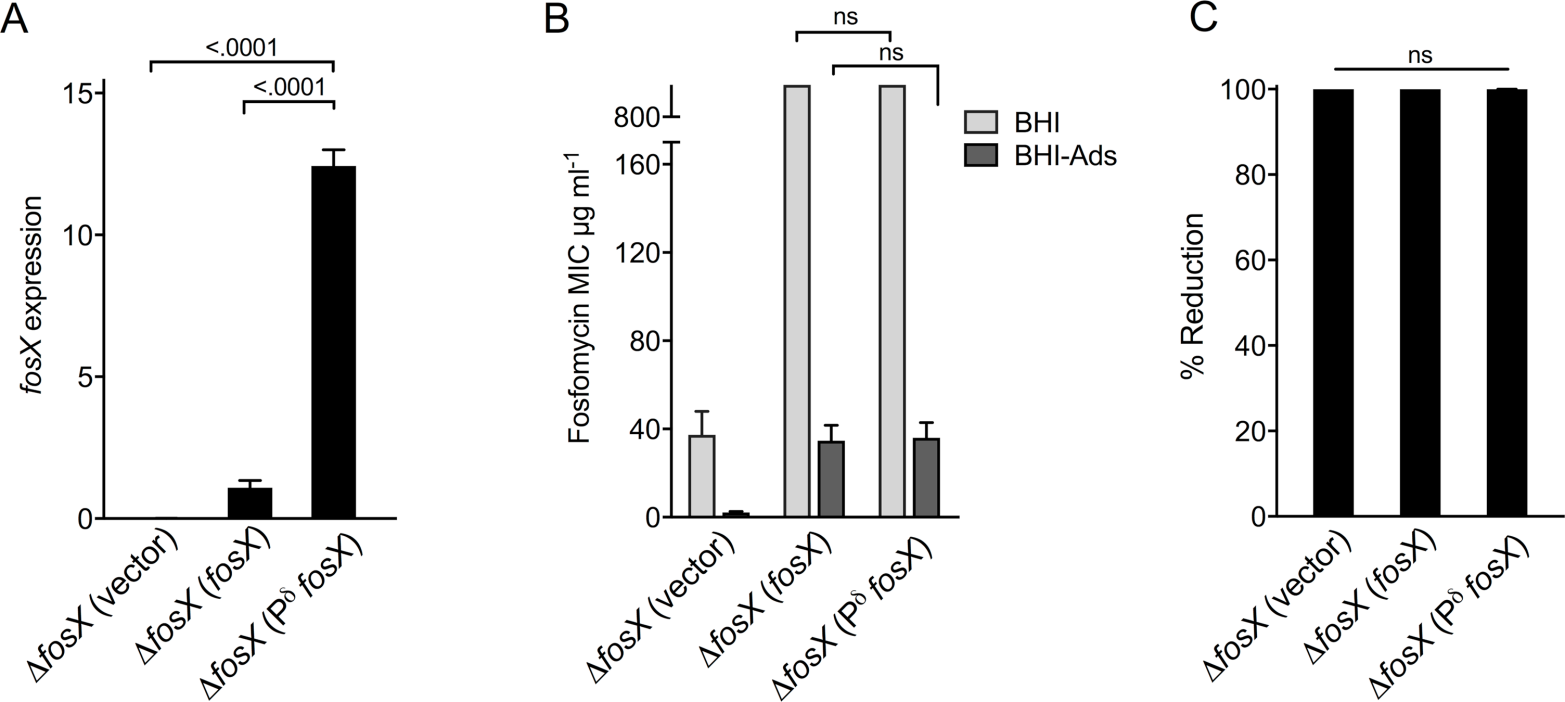
Fosfomycin susceptibility is not affected by *fosX* overexpression. *L*. *monocytogenes* Δ*fosX* was complemented with the integrative plasmid pPL2 carrying the *fosX* gene expressed from its native P^lmo1703^ promoter (*fosX*) or the strong Pδ promoter (P^δ^*fosX*), or empty vector as a control. **(A)** RT-QPCR analysis of *fosX* transcription in BHI grown bacteria. **(B)** Fosfomycin MICs determined in BHI and charcoal-supplemented BHI (BHI-Ads). **(C)** Percent reduction of intracellular bacteria in RAW 264.7 macrophages in response to fosfomycin (180 μg/ml) relative to untreated infected cells. Determined at 23 h of infection. Mean ± SEM of at least three independent experiments. Relevant *P* values are indicated (one-way and two-way [panel B] ANOVA with Šídák multicomparison test); ns, not significant.

We finally examined the impact of the potential occurrence of *fosX* overexpression mutants on the *L*. *monocytogenes* fosfomycin phenotype. To approximate this, we expressed the *fosX* gene in P14Δ*fosX* under the control of a strong constitutive gram-positive promoter (Pδ [44]). As shown in Fig 9A, Pδ drove *fosX* expression to significantly higher levels compared to an equivalent construct with the native P^lmo1703^ promoter from which *fosX* is expressed in *L*. *monocytogenes* [29]. Fosfomycin susceptibility of the two constructs and mock-complemented Δ*fosX* (control) was analyzed *in vitro* in BHI (PrfA”Off”) and BHI-Ads as well as infected RAW 264.7 macrophages (PrfA “On”). The data show that overexpression of *fosX* neither modified the *in vitro* fosfomycin MIC (specifically in BHI-Ads, *P* = 0.999) (Fig 9B) nor significantly affected bacterial susceptibility in infection conditions (percent reduction of intracellular population respect to untreated control between 99.97 and 99.99% for the three strains) (*P* = 0.115) (Fig 9C). Thus, promoter mutations leading to increased *fosX* expression are unlikely to result in adaptive fosfomycin resistance due to the epistatic effect of *prfA*/*hpt* taking prevalence even when *fosX* is overexpressed. Why *fosX* overexpression does not result in increased levels of fosfomycin resistance may be related to a variety of reasons, including gene post-transcriptional control or stoichiometric limitations to enzyme activity, and remains to be investigated.

## Discussion

Experimental studies showing that antimicrobial resistance may not only carry fitness costs but also enhance bacterial performance *in vivo* [45, 46] have prompted a renewed interest in understanding the complex relationships between resistance and virulence [47–49]. A direct link is obvious when the corresponding determinants are carried on the same mobile genetic element [47, 50, 51]. Other illustrative examples include the efflux pumps that serve a dual role in both resistance and virulence [52, 53], or when there is co-ordinate modulation or crosstalk between resistance and virulence gene regulatory networks [49, 51, 54, 55]. Most often, however, the connection is subtler, detected at population level through epidemiological [56–58] or genome-wide co-evolution studies [59], likely involving gene-gene interactions [47, 56], and little is known about the precise underlying molecular mechanisms [48]. In this study, we report a compelling example of gene interaction where the resistance phenotype conferred by a *Listeria* core genome trait is modified by virulence genes specifically present in the genomic background of the pathogenic species *L*. *monocytogenes*, strongly affecting the susceptibility to an antibiotic.

Independently, the loci involved, the fosfomycin resistance gene *fosX* and the virulence genes *hpt* and *prfA*, are bacterial performance enhancers in the specific conditions for which they presumably evolved in *Listeria*, i.e. exposure to naturally occurring phosphonic acid metabolites or the intracellular compartment of an animal host, respectively. However, in the (man-made) event of *L*. *monocytogenes* simultaneously confronting fosfomycin and the host, the effect of *prfA* and *hpt* is dominantly deleterious and overrides the beneficial effect of *fosX* (Fig 5). In such circumstances, *hpt* and its regulatory gene *prfA* “stand above” and “stop” the fosfomycin resistance phenotype conferred by *fosX* in an archetypal example of epistasis [60, 61], to our knowledge the first to be dissected in molecular mechanistic detail between virulence and resistance genes in a bacterial pathogen.

Gene-gene epistatic interactions are thought to play a critical role in modulating the phenotypic expression of antibiotic resistance and its impact on microbial fitness, and thereby in the evolution of resistance [62–67]. Our findings extend this notion to the relationships between resistance and virulence, two key specific, clinically relevant pathogen traits. In the particular case described herein, the characterized virulence-resistance gene interplay renders *L*. *monocytogenes* susceptible to fosfomycin *in vivo* during infection although carrying the *fosX* gene, which otherwise confers high levels of resistance to the antibiotic.

Our data indicate that, due to the epistatic effect of the virulence genes *prfA* and *hpt* (which cannot be reversed by *fosX* overexpression), or even naturally occurring spontaneous *fosX* mutations, the vast majority of *L*. *monocytogenes* strains are expected to be fully susceptible to fosfomycin in clinical conditions. Together with our earlier finding that acquired fosfomycin resistance is mostly due to mutations in the virulence genes *hpt* and *prfA*, and hence counterselected during infection [17], this report provides the rationale underpinning the use of fosfomycin against a nominally intrinsically resistant *L*. *monocytogenes*.

The currently recommended treatment for listeriosis consists in a prolonged course of amoxicillin or ampicillin at high doses, often in combination with gentamicin [1, 2, 9, 68]. However, aminopenicillins cannot be administered in case of allergy to β-lactams, and gentamicin has a poor intracellular penetration, does not cross the BBB efficiently, and is nephrotoxic or ototoxic [10, 69, 70]. Due to its bactericidal activity and synergistic action with many antimicrobials including β-lactams, aminoglycosides, meropenem, linezolid, daptomycin and vancomycin, the “old” antibiotic fosfomycin [14] is currently resurging as a therapeutic option for the treatment of critically ill patients with invasive or systemic bacterial infections [12, 13, 71]. Rapid, effective bactericidal action is paramount in neuromeningeal, bacteremic or materno-fetal listeriosis to limit disease severity, relapses and fatal outcomes [2, 9, 70, 72, 73]. With a well-established safety record, low plasma protein binding, excellent intracellular penetration, and superior entry through the BBB and placental barrier than β-lactams [11–13, 74], intravenous fosfomycin may prove highly beneficial in the combination therapy of listeriosis. Beyond its fundamental implications, the mechanistic characterization of the epistatic interaction between resistance and virulence genes reported here has, therefore, a potential direct application in the treatment of a life-threatening infectious disease.

## Materials and methods

### Bacteria and culture conditions

The bacterial strains used in this study are listed in S2 and S3 Tables. *L*. *monocytogenes* isolates in Fig 8 are of diverse origins (clinical human and animal, food, environment) and phylogenomic clades, including lineages I, II and III); they were sourced from Institut Pasteur’s *Listeria* collection or JV-B laboratory’s isolate collection. *Listeria* were grown in Brain-Heart Infusion (BHI, Porcine, BD Difco) and *Escherichia coli* in Luria-Bertani (LB, Sigma) media (supplemented with 1% agar w/v and/or antibiotics as appropriate) at 37 °C unless otherwise stated. The PrfA regulon was activated *in vitro* by supplementing BHI with the adsorbents, activated charcoal (0.5% w/v, Merck) or Amberlite XAD-4 (1% w/v, Sigma), as previously described [37] (referred to as “BHI-Ads”).

### Bioinformatic analyses

The *L*. *monocytogenes* genome dataset (*n* = 1,696 sequences) was scanned for the presence of *fosX* (*lmo1702*) using the BLASTn algorithm [75] implemented in BIGSdb v.1.16 platform [76] as described in ref. [24], with minimum nucleotide identity of 70%, alignment length coverage of 70% and word size of 10. General homology searches were carried out using BLASTP with default parameters.

### *fosX* constructs

*L*. *monocytogenes* P14 *fosX* deletions were made by allelic exchange using the thermosensitive shuttle vector pMAD [77] as previously described [7]. A DNA fragment containing an in-frame Δ*fosX* allele comprising the last five 5’- and 3’-terminal codons of the wild-type gene was prepared by splicing overlap extension PCR using oligonucleotides fosXDM-1 NcoI and fosXDM-4 BamHI (S4 Table), as previously described [5]. The PCR product was inserted into pMAD using the NcoI and BamHI restriction sites and the resulting pMADΔ*fosX* plasmid (S2 Table) was transformed into *L*. *monocytogenes*. Allelic exchange was selected at the vector-non-permissive temperature of 42 °C as described in [19] and verified by PCR and DNA sequencing. For complementation, a single copy of the *fosX* gene from P14 was inserted into the *L*. *monocytogenes* chromosome under the control of its native P^lmo1703^ promoter [29] (S1 Fig) using the integrative vector pPL2 [78] (S2 Table). The P^lmo1703^:*fosX*^P14^ fusion was constructed by ligating two PCR products, one containing the 5’ UTR region of *lmo1703* including the promoter, the other the coding sequence of *fosX* from strain P14, using the NdeI site included in the 5’ tails of oligonucleotides Plmo1703-NdeI-R and fosX-ATG-NdeI-F (S4 Table). The resulting ligation product was inserted in the multicloning site (MCS) of pPL2 using SacI-SalI sites (plasmid pPLP^lmo1703^:*fosX*^P14^, S2 Table). For *fosX* overexpression, the *fosX* gene plus 45 bp upstream was placed under the control of the strong constitutive Pδ promoter from the streptococcal pSM19035 plasmid partitioning gene δ [44] and inserted into pPL2’s MCS using SpeI-BamHI sites (plasmid pPLPδ:*fosX*^P14^, S2 Table). pPL2 integrants were selected in BHI containing 7.5 μg/ml chloramphenicol and confirmed by PCR and DNA sequencing. The *L*. *innocua fosX* gene was knocked out by plasmid insertional mutagenesis. A 363-bp internal *fosX* gene fragment was PCR-amplified (see oligonucleotides in S4 Table) and ligated into the BamHI-EcoRI sites of the thermosensitive vector pLSV1 [79]. The resultant pLSV-*fosX*^*Li*^ plasmid was introduced into *L*. *innocua* CLIP11262 (S2 Table) and the recombinant clones were selected at 42 °C in BHI containing 5 μg/ml erythromycin. *L*. *innocua* wild-type *fosX* revertants were obtained by serial passage in BHI without erythromycin and selected on 150 μg/ml fosfomycin, a concentration that is inhibitory when the *fosX* gene is inactivated (see Fig 2). Correct plasmid insertion and subsequent plasmid loss and restoration of the wild-type *fosX* allele was confirmed by PCR and DNA sequencing. Restriction enzymes were obtained from New England Biolab and high-fidelity PfuUltra II Hotsart DNA polymerase from Agilent.

### Fosfomycin susceptibility testing

Fosfomycin MICs were determined by the Etest strip method (bioMérieux) after 24 h incubation at 37 °C as previously described [17].

### Characterization of PrfA phenotype

The functional status of PrfA was determined using the activities of three PrfA-regulated virulence determinants used as reporters (*hly* encoding the hemolysin listeriolysin O [LLO], *plcB* encoding the phosphatidyl-choline preferring phospholipase C/lecithinase PlcB, and *hpt* encoding the Hpt sugar phosphate permease), as previously described [7, 17, 37]. Briefly, LLO activity was determined by the halo of hemolysis around *Listeria* colonies in sheep blood agar (SBA, bioMérieux); PlcB activity was determined by the halo of precipitation in BHI supplemented with 10% of an egg yolk suspension, prepared by dispersing one egg yolk in 100 ml of sterile saline solution; and Hpt activity was determined using a sugar acidification test in phenol red base broth (Oxoid) supplemented with 10 mM glucose-1-phosphate after 24 h incubation (G1P, Sigma). PlcB and Hpt tests were carried out also with and without supplementation with 0.5% activated charcoal. Using this panel of tests, the *L*. *monocytogenes* wild-type phenotype is characterized by: (i) weak hemolysis in SBA (confined to the area beneath the colonies), (ii) negative lecithinase reaction and G1P utilization in non-charcoal-supplemented media; and (iii) strong lecithinase and G1P acidification in charcoal-supplemented medium. A PrfA* phenotype is characterized by (i) strong hemolysis in SBA and (ii) strong lecithinase and positive G1P in non-charcoal-supplemented medium.

### Competition assays

Inocula for the competition assays were prepared from *L*. *monocytogenes* overnight BHI cultures shaken at 200 rpm until OD_600_ ≈1. Bacteria were collected by centrifugation, washed twice in PBS, suspended in 10% glycerol PBS and stored at -80°C in 100μl aliquots. Viable numbers of each P14 and P14Δ*fosX* strains in the frozen alliquots were determined by plate counting and suitable amounts mixed to prepare a 1:1 suspension. For the *in vitro* competition assays in BHI, five ml of fresh culture medium were seeded with 40 μl of the 1:1 suspension of P14 and P14Δ*fosX* (≈2×10^6^ CFU/ml total bacteria) and growth was monitored during incubation with orbital shaking (200 rpm) using an automated plate reader (Omega apparatus, BMG Labtech), as previously described [7]. For assays in infected cells, mycoplasma-free RAW 264.7 mouse macrophage monolayers (sourced from ATCC) grown in Dulbecco modified Eagle medium supplemented with 10% fetal bovine serum (Gibco) (DMEM) were inoculated with the 1:1 bacterial suspension at a multiplicity of infection of 10 and intracellular bacterial proliferation monitored using a gentamicin protection assay, as previously described [5]. The competing wild-type and Δ*fosX* bacteria were enumerated by differential plating in BHI and BHI supplemented with 100 μg/ml fosfomycin (inhibitory for *L*. *monocytogenes* Δ*fosX*, MIC 45.3 μg/ml; Fig 2). The genotype of the bacterial strains was further confirmed by PCR in 50 randomly selected colonies using oligonucleotides fosXDM-5 and fosXDM-6 (S4 Table). The competitive index (CI) was calculated using the formula CI = (test/reference log CFU ratio at *t* = n)/(test/reference log CFU ratio at *t* = 0).

### Intracellular susceptibility assays

The effect of fosfomycin on intracellular *L*. *monocytogenes* was determined as previously described [17]. Briefly, 80–90% confluent monolayers of RAW 264.7 macrophages or HeLa epithelial cells (ATCC) were infected at 10:1 or 25:1 multiplicity, respectively, and centrifuged at 900 rpm to synchronize infection. After 30 min of incubation, infected monolayers were washed with PBS to remove extracellular bacteria and incubated in DMEM containing 100 μg/ml gentamicin for 30 min (*t* = 0). At 40 min after *t* = 0, the medium was changed to 25 μg/ml gentamicin with or without 180 μg/ml fosfomycin.

### Gene expression analysis

Total *L*. *monocytogenes* RNA was extracted from mid-exponential cultures (OD_600_≈ 0.2–0.3 for BHI media) or intracellular bacteria (*t* = 4 h) using RNease mini kit (Qiagen). RNA samples were reverse transcribed using ImProm-II^TM^ reverse transcription system (Promega) and specific cDNAs quantified by real-time PCR (RT-QPCR) as previously described [5] using Step One Plus real-time PCR apparatus and Step One V2.3 software (Applied Biosystems). The PCR signal was monitored using TaqMan probes for the PrfA-regulated genes *hpt* and *actA*, and Power SYBR Green master mix (Applied Biosystems) and gene-specific primers for *fosX*. Transcription values of the target genes were normalized using the housekeeping genes *rpoB* and *ldh*. Fold-changes in fosX expression were determined by the 2^–ΔΔCT^ method. The oligonucleotides used are shown in S4 Table.

### Statistical analysis

Statistical significance was analyzed using Prism 7.0 software (GraphPad Software Inc., San Diego, CA). The specific tests used are indicated in the figure legends.

### Accession codes

Sequence data obtained in this study are available from the European Nucleotide Archive (ENA) under accession no. LT795753, LT795754, LT795755, LT795756, LT795757, LT795758, LT795759, LT795760, LT795761, LT795762.

## Supporting information

S1 Fig Genetic and transcriptional organization of *L*. *monocytogenes fosX* locus. Based on detailed transcription unit mapping of the *L*. *monocytogenes* EGDe genome by massive strand-specific cDNA sequencing [29]. Transcription start site and terminator are indicated. Color codes of genes as in Fig 1C.
(TIF)

S2 Fig Genetic structure of *fosX* locus in different bacteria. Schematic representation comparing the genomic regions around the *fosX* gene (in black) in a selection of bacteria from different phyla showing highest FosX amino acid sequence similarity to *Listeria*. Putative functions encoded are indicated; HP, hypothetical protein. Note that the *fosX* region in each bacterial species has a different genetic structure (color codes of *Listeria* genes as in Fig 1C, non-matching genes in other species are shown in grey). Genome sequences analyzed (NCBI accession nos.): *Pelosinus fermentans* A11 (NZ_AKVM01000112.1), *Brevibacillus thermoruber* PM1(NZ_JQMH01000006.1), *Enterococcus silesiacus* DSM 22801 (NZ_JXLC01000010.1), *Bacillus patagoniensis* DSM 16117 (NZ_KV917377.1), *Clostridium botulinum* F634 (CP013707.1), *M*. *loti* NZP2014 (LYTJ01000024.1), *D*. *hafniense* TCP-A (NZ_KB900391.1), *Sulfitobacter mediterraneus* DSM 12244 (NZ_QBKU01000002.1), *Brucella melitensis* S66 (NZ_AHWB01000021.1), *Synechococcus* sp. PCC 6312 (NC_019680.1). Genes not at exact scale.
(TIF)

S3 Fig Expression of *hpt* gene in *L*. *monocytogenes fosX* mutants with “slow-positive” Hpt phenotype. Transcription analysis of *hpt* gene and control PrfA-regulated *actA* gene determined by RT-QPCR in PAM 3393 and PAM 3415 mutants (see S3 Table) and wild-type *L*. *monocytogenes* P14 grown in BHI (PrfA “Off”). Mean ± SEM of four independent experiments in duplicate. One-way ANOVA with Dunnett´s multitple comparison tests; ns, not significant.
(TIF)

S4 Fig Structural analysis of spontaneous *fosX*^128stop^ mutation. A premature stop codon at triplet 128 (of 133) of the *L*. *monocytogenes fosX* gene was present in seven out of nine constitutively susceptible *L*. *monocytogenes* human clinical isolates tested (see text). Complementation analysis showed that the *fosX*^128stop^ allele is non-functional, indicating that the truncated FosX product is either inactive or unstable. The three-dimensional structure of the *L*. *monocytogenes* FosX metalloenzyme dimer [22] (PDB 2P27; from serovar 4b strain ATCC19115, with FosX of P14 sequence type, see S3 Table) is shown with polypeptide chains in green and gray. The C-terminal region missing in the truncated FoxX polypeptide (residues 128–133, in blue) appears to play a critical role in stabilizing the cup-shaped metal coordination/catalytic site of the enzyme [22] via hydrophobic interactions with residues from the three-stranded antiparallel β-sheet domain in the opposite protomer (Leu128 with Ile31’, Tyr32’, Phe46’ and, indirectly via Leu124, Trp53’; Tyr131 with Tyr32’; in sphere representation except Ile31’). Mn(II) ions are in magenta, the bound sulfate ion expected to indicate the position of the phosphonate group of fosfomycin is in stick representation with colored atoms.
(TIF)

S1 Table Distribution of *fosX* in *Listeria* spp. FosX orthologs (73–91% identity, 100% coverage over 133 residues) are encoded in all *Listeria sensu stricto* species except *L*. *seeligeri*. FosX paralogs (63–66% amino acid sequence identity, i.e. similar to the level of homology with FosX proteins from more distantly related bacteria) are encoded in a different chromosomal location in the *Listeria sensu lato* “*Paenilisteria*” clade except *L*. *grandensis* (in *L*. *cornellensis* and *L*. *rocourtiae* the gene is truncated). More distant paralogs are encoded in *Murraya grayi* (55% identity, truncated) and *Listeria sensu lato* “*Mesolisteria*” clade. (23–55%). FosX homologs from other *Firmicutes* and α-*Proteobacteria* bacteria originally described in ref. [23] are included for reference.
(PDF)

S2 Table Strains and plasmids. Used in the genetic analysis of the role of *fosX*, *hpt* and *prfA* in *Listeria* fosfomycin susceptibility.
(PDF)

S3 Table Analysis of PrfA phenotype and *fosX* genotype of *L*. *monocytogenes* clinical isolates constitutively susceptible to fosfomycin (BHI MIC ≤64 μg/ml). Human isolates with wild-type fosfomycin susceptibility pattern (resistant in BHI and susceptible in BHI-Ads), including a selection of strains showing lowest MICs in the resistance range in BHI, were also sequenced as controls. Reference strains: P14, P14 prfA* and EGDe. Two other well-characterized *L*. *monocytogenes* strains (10403S and CLIP 80459) were also analyzed.
(PDF)

S4 Table Main oligonucleotides used in this study. Relevant restriction sites are underlined.
(PDF)
