# Correction: Epistatic control of intrinsic resistance by virulence genes in *Listeria*

**DOI:** 10.1371/journal.pgen.1007727

**Published:** 2018-10-15

**Authors:** Mariela Scortti, Lei Han, Sonsiray Alvarez, Alexandre Leclercq, Alexandra Moura, Marc Lecuit, Jose Vazquez-Boland

There is an error in affiliation 3. Affiliation 3 should be: Institut Pasteur, Biology of Infection Unit, INSERM U1117 and National Reference Centre / WHO Collaborating Centre for Listeria, Paris, France.

In the Author Contributions section, Marc Lecuit should be listed as one of the persons who contributed to “Formal analysis”.

The following information is missing from the Funding section: ML’s laboratory is funded by Institut Pasteur, Inserm and Santé Publique France.
