# Supplementary figures and images for: Epistatic control of intrinsic resistance by virulence genes in Listeria
Source: PLoS Genet. 2018 Sep 4;14(9):e1007525. doi: 10.1371/journal.pgen.1007525 (PMC6122793; doi:10.1371/journal.pgen.1007525)

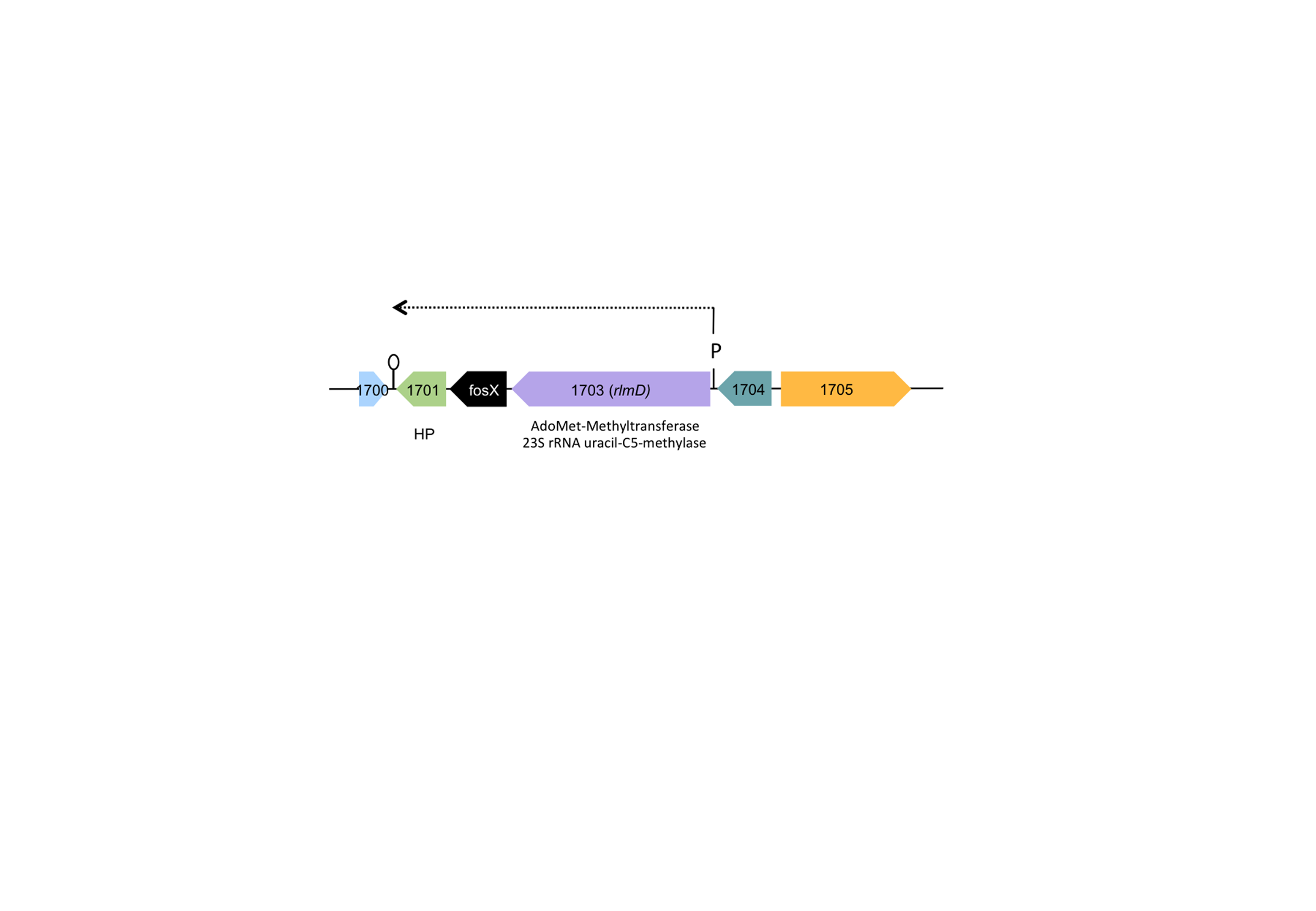

Supplement: S1 Fig — Based on detailed transcription unit mapping of the L. monocytogenes EGDe genome by massive strand-specific cDNA sequencing [29]. Transcription start site and terminator are indicated. Color codes of genes as in Fig 1C. (TIF) [file pgen.1007525.s001.tif]

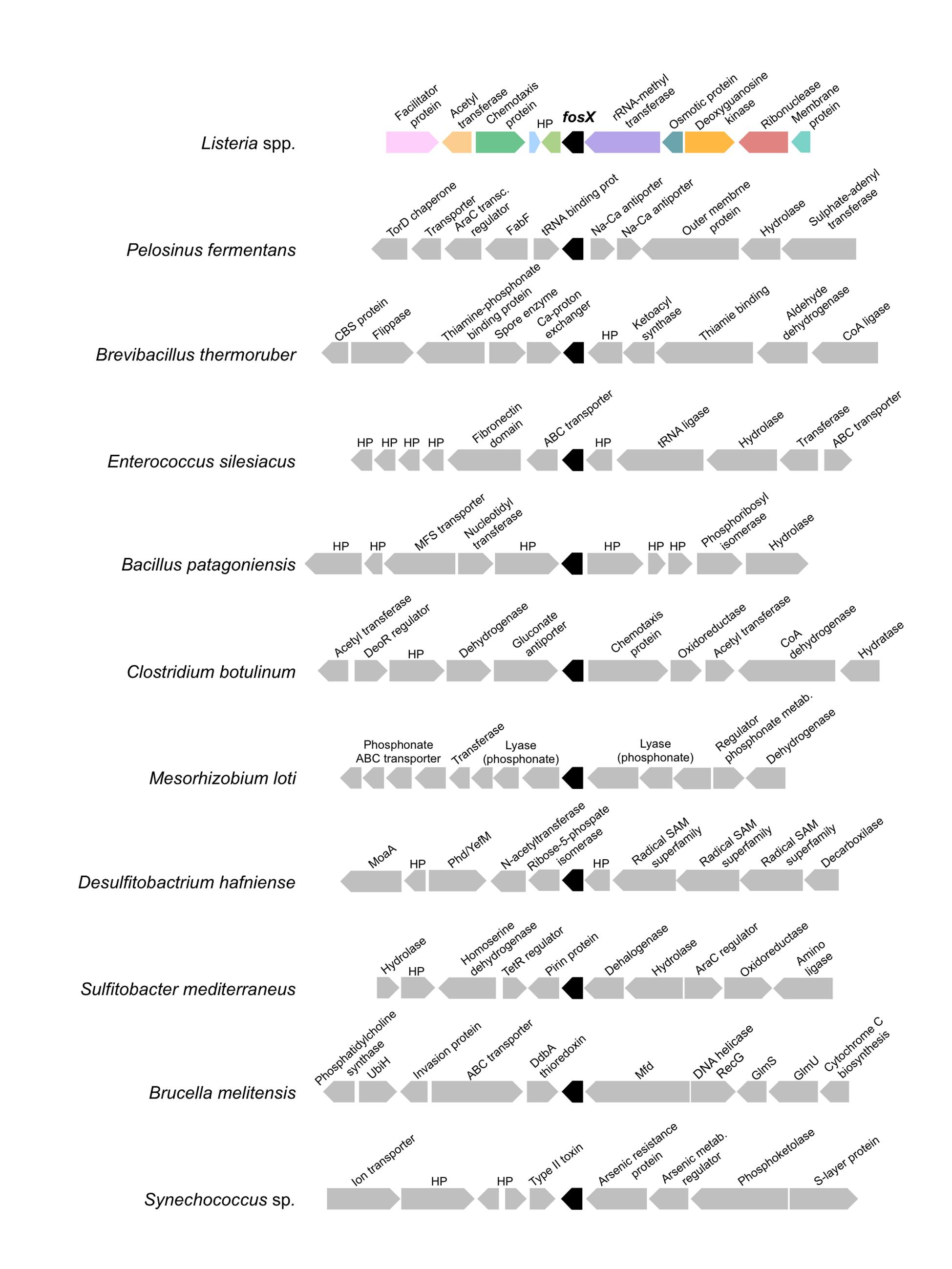

Supplement: S2 Fig — Schematic representation comparing the genomic regions around the fosX gene (in black) in a selection of bacteria from different phyla showing highest FosX amino acid sequence similarity to Listeria. Putative functions encoded are indicated; HP, hypothetical protein. Note that the fosX region in each bacterial species has a different genetic structure (color codes of Listeria genes as in Fig 1C, non-matching genes in other species are shown in grey). Genome sequences analyzed (NCBI accession nos.): Pelosinus fermentans A11 (NZ_AKVM01000112.1), Brevibacillus thermoruber PM1(NZ_JQMH01000006.1), Enterococcus silesiacus DSM 22801 (NZ_JXLC01000010.1), Bacillus patagoniensis DSM 16117 (NZ_KV917377.1), Clostridium botulinum F634 (CP013707.1), M. loti NZP2014 (LYTJ01000024.1), D. hafniense TCP-A (NZ_KB900391.1), Sulfitobacter mediterraneus DSM 12244 (NZ_QBKU01000002.1), Brucella melitensis S66 (NZ_AHWB01000021.1), Synechococcus sp. PCC 6312 (NC_019680.1). Genes not at exact scale. (TIF) [file pgen.1007525.s002.tif]

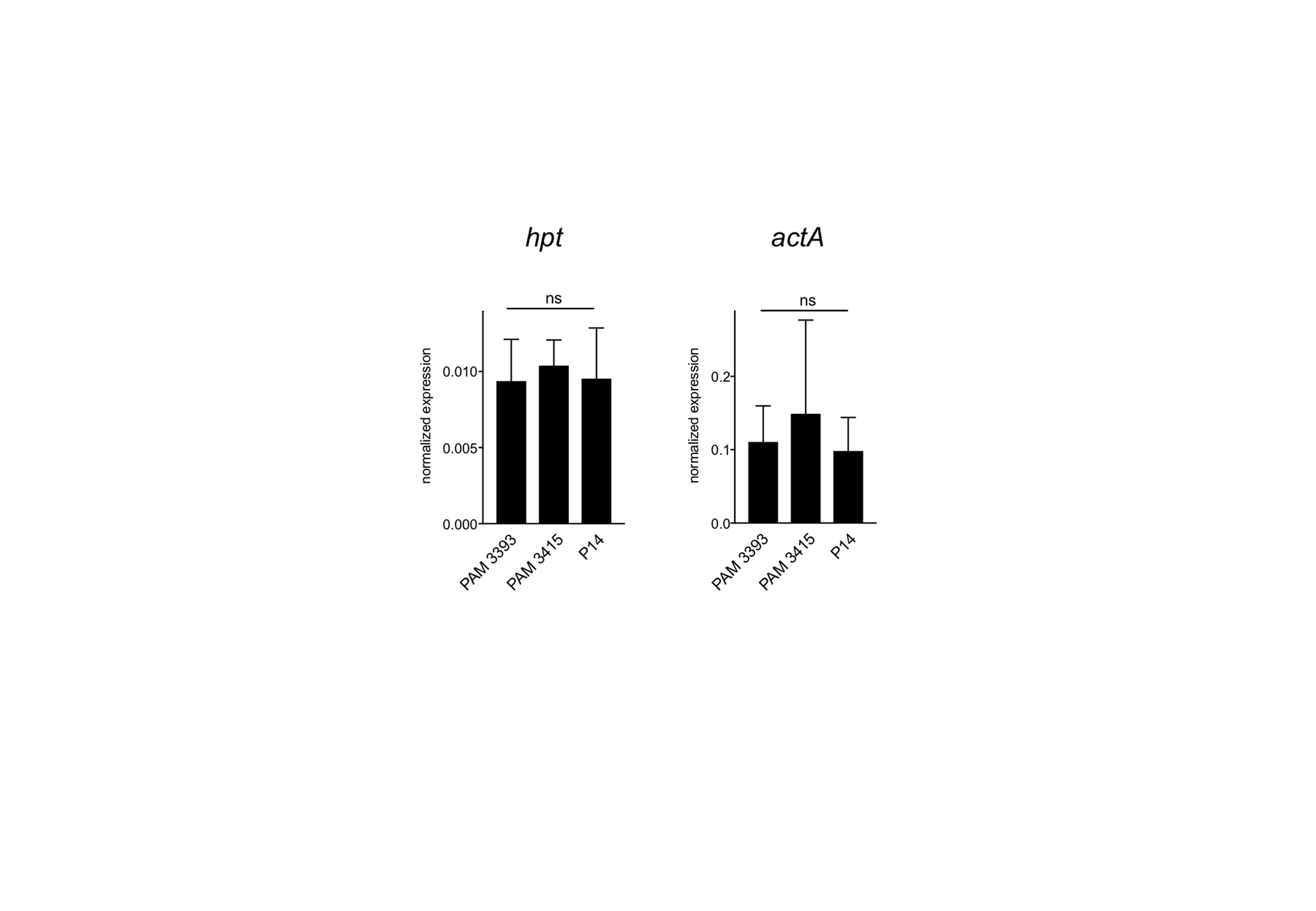

Supplement: S3 Fig — Transcription analysis of hpt gene and control PrfA-regulated actA gene determined by RT-QPCR in PAM 3393 and PAM 3415 mutants (see S3 Table) and wild-type L. monocytogenes P14 grown in BHI (PrfA “Off”). Mean ± SEM of four independent experiments in duplicate. One-way ANOVA with Dunnett´s multitple comparison tests; ns, not significant. (TIF) [file pgen.1007525.s003.tif]

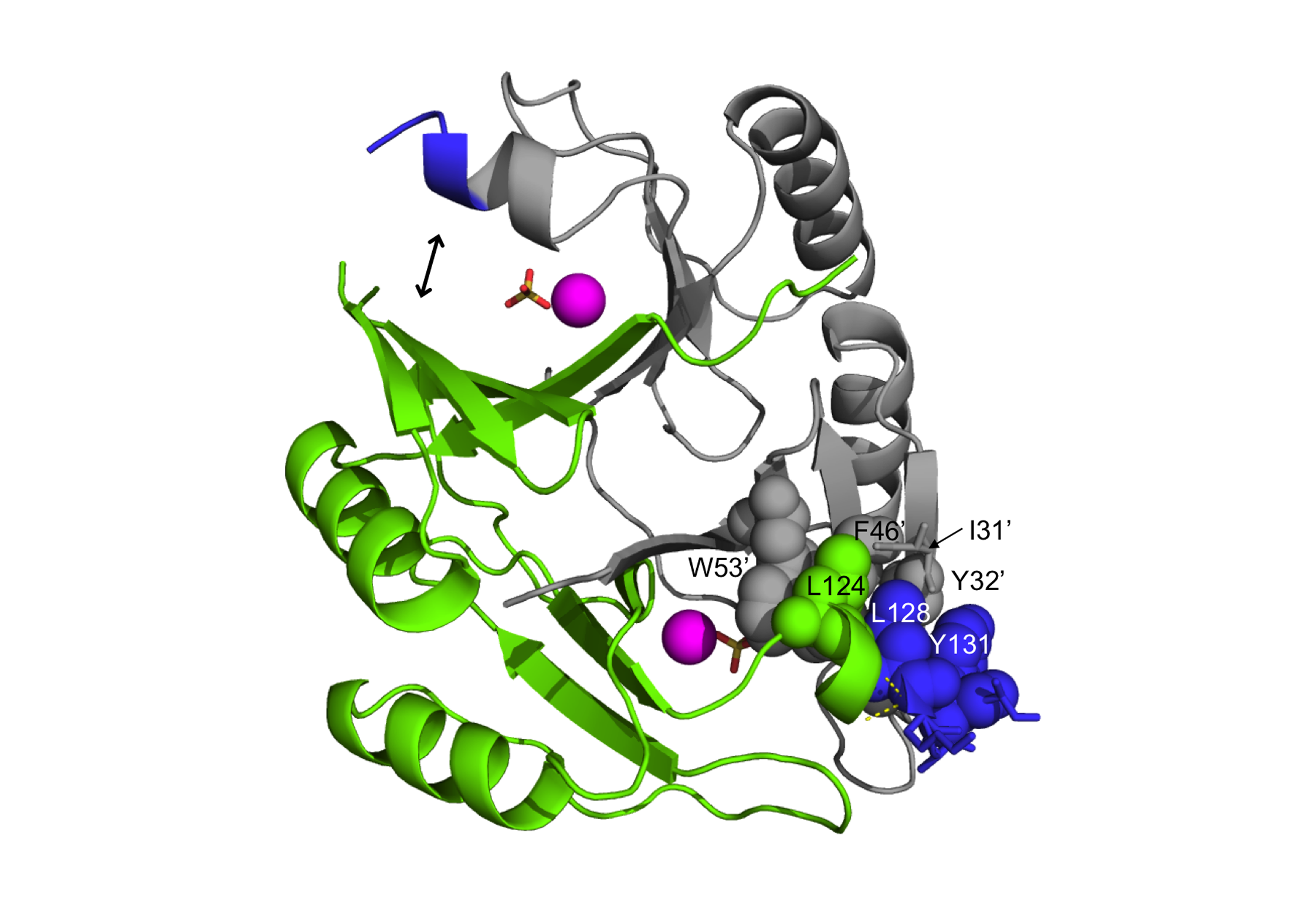

Supplement: S4 Fig — A premature stop codon at triplet 128 (of 133) of the L. monocytogenes fosX gene was present in seven out of nine constitutively susceptible L. monocytogenes human clinical isolates tested (see text). Complementation analysis showed that the fosX128stop allele is non-functional, indicating that the truncated FosX product is either inactive or unstable. The three-dimensional structure of the L. monocytogenes FosX metalloenzyme dimer [22] (PDB 2P27; from serovar 4b strain ATCC19115, with FosX of P14 sequence type, see S3 Table) is shown with polypeptide chains in green and gray. The C-terminal region missing in the truncated FoxX polypeptide (residues 128–133, in blue) appears to play a critical role in stabilizing the cup-shaped metal coordination/catalytic site of the enzyme [22] via hydrophobic interactions with residues from the three-stranded antiparallel β-sheet domain in the opposite protomer (Leu128 with Ile31’, Tyr32’, Phe46’ and, indirectly via Leu124, Trp53’; Tyr131 with Tyr32’; in sphere representation except Ile31’). Mn(II) ions are in magenta, the bound sulfate ion expected to indicate the position of the phosphonate group of fosfomycin is in stick representation with colored atoms. (TIF) [file pgen.1007525.s004.tif]
