# Supplementary material for: Epistatic control of intrinsic resistance by virulence genes in Listeria
Source: PLoS Genet. 2018 Sep 4;14(9):e1007525. doi: 10.1371/journal.pgen.1007525 (PMC6122793; doi:10.1371/journal.pgen.1007525)
Supplement: S1 Table — FosX orthologs (73–91% identity, 100% coverage over 133 residues) are encoded in all Listeria sensu stricto species except L. seeligeri. FosX paralogs (63–66% amino acid sequence identity, i.e. similar to the level of homology with FosX proteins from more distantly related bacteria) are encoded in a different chromosomal location in the Listeria sensu lato “Paenilisteria” clade except L. grandensis (in L. cornellensis and L. rocourtiae the gene is truncated). More distant paralogs are encoded in Murraya grayi (55% identity, truncated) and Listeria sensu lato “Mesolisteria” clade. (23–55%). FosX homologs from other Firmicutes and α-Proteobacteria bacteria originally described in ref. [23] are included for reference. (PDF) [file pgen.1007525.s005.pdf]

S1 Table. Distribution of *fosX* in *Listeria* spp. FosX orthologs (73-91% identity, 100% coverage over 133 residues) are encoded in all *Listeria* "*sensu stricto*" species except *L. seeligeri*. FosX homologs (63-66% amino acid sequence identity, i.e. similar to the level of homology with FosX proteins from more distantly related bacteria) are encoded in a different chromosomal location in the "*Paenilisteria*" clade except *L. grandensis* (in *L. cornellensis* and *L. rocourtiae* the gene is truncated). More distant homologs are encoded in *Murraya grayi* (55% identity, truncated) and "*Mesolisteria*" spp. (23-55%). The *fosX*-like genes in the "*Paenilisteria*", "*Mesolisteria*" and *Murraya* clades probably represent evolutionary paralogs. FosX homologs from other *Firmicutes* and α-*Proteobacteria* bacteria originally described in ref. [23] are included for reference.

Species	Classification [a]	Strain	*Lm* serovar, lineage	NCBI RefSeq / Accession no.	Aa identity (%)	Coverage (%)	Amino acids
L. monocytogenes	*Listeria* "*sensu stricto*"	EGDe	1/2a, LII	WP_010989789.1	100 [b]	100	133
	"	P14	4b, LI	LT795753	92 [b]	100	133
	"	10403S	1/2a, LII	WP_014600911.1	95 [b]	100	133
	"	SLCC 2755	1/2b, LI	WP_003726635.1	92	100	133
	"	SLCC 2372	1/2c, LII	WP_010989789.1	100	100	133
	"	L99	4a, LIII	WP_012581185.1	93	100	133
	"	HCC23	4a, LIII	WP_012581185.1	93	100	133
	"	F2365	4b, LI	WP_003726635.1	92	100	133
	"	H7858	4b, LI	WP_003726635.1	92	100	133
	"	SLCC 2376 [b]	4c, LIII	WP_003730577.1	96 [b]	100	133
	"	SLCC 2378	4e, LI	WP_003726635.1	92	100	133
	"	ATCC 19117	4d, LI	WP_003726635.1	92	100	133
	"	SLCC 7179	3a, LII	WP_010989789.1	100	100	133
	"	SLCC 2479	3c, LII	WP_010989789.1	100	100	133
	"	SLCC 2540	3b, LI	WP_003726635.1	100	100	133
L. innocua	"	CLIP11262		WP_003762632.1	89	100	133
L. marthii	"	FSL S40-120		WP_010989789.1	91	100	135
L. welshimeri	"	SLCC5334		WP_011702500.1	89	100	133
L. ivanovii	"	PAM 55		WP_014093062.1	73	100	133
L. seeligeri	"	SLCC3954		–	–	–	–
L. weihenstephanensis	"*Paenilisteria*"	FSL R9-0317		WP_036061641.1	65	97	133
L. riparia	"	FSL S10-1204		WP_036101082.1	63	97	133
L. cornellensis	"	FSL F6-969		EUJ2945.1	66	57	79
L. rocourtiae	"	FSL F6-920		EUJ44943	63	53	73
L. booriae	"	FSL A5-0281		WP_036087766.1	63	97	133
L. newyorkensis	"	FSL A5-0209		WP_030689544.1	66	99	133
L. grandensis	"	FSL F6-0971		–	–	–	–
L. grayi	*Murraya*	FSL F6-1183		WP_036103975.1	55	50	76
L. fleischmannii	"*Mesolisteria*"	FSL S10-1203		WP_036061826.1	23	48	99
L. aquatica	"	FSL S10-1188		WP_036073138	34	43	100
L. floridensis	"	FSL S10-1187		WP_036098293	55	30	130
Mesorhizobium loti	*Rhizobiales*	NZP2014		WP_064991938.1	63	98	139
Brucella melitensis	*Rhizobiales*	S66		WP_004687281.1	58	98	139
Clostridium botulinum	*Clostridiales*	Kyoto		WP_012705368	56	98	137
Desulfitobacterium	*Clostridiales*	TCP-A		WP_018213323.1	63	98	137

[a] Classification of *Listeria* based on phylogenomic clades as per ref. [25].

[b] The FosX amino acid sequence differs in 10 positions between strains EGDe (serovar 1/2a) and P14 (serovar 4b), defining two major lineage-related sequence types; see S3 Table. There are exceptions, for example FosX from strain 10403S (serovar 1/2a, lineage II) differs by five amino acid substitutions with the EGDe sequence. *L. monocytogenes* lineage III strains define a third FosX sequence type, in turn with internal sequence polymorphisms. See Fig 1.
