# Supplementary material for: Epistatic control of intrinsic resistance by virulence genes in Listeria
Source: PLoS Genet. 2018 Sep 4;14(9):e1007525. doi: 10.1371/journal.pgen.1007525 (PMC6122793; doi:10.1371/journal.pgen.1007525)
Supplement: S2 Table — Used in the genetic analysis of the role of fosX, hpt and prfA in Listeria fosfomycin susceptibility. (PDF) [file pgen.1007525.s006.pdf]

S2 Table. Strains and plasmids. Used in the genetic analysis of the role of *fosX*, *hpt* and *prfA* in *Listeria* fosfomycin susceptibility.

Strain/plasmid	Description	Source [reference]	Internal collection no.
L. monocytogenes			
P14	Wild-type human clinical isolate, neuromeningits, serovar 4b	Our laboratory [38]	PAM 14
P14Δ*prfA*	In-frame *prfA* deletion mutant	Our laboratory [17,19]	PAM 373
P14Δ*hpt*	In-frame *hpt* deletion mutant	Our laboratory [17,19]	PAM 376
P14Δ*fosX*	In-frame *fosX* deletion mutant	This study	PAM 3641
P14Δ*fosX*Δ*hpt*	In-frame double *hpt* and *fosX* deletion mutant	This study	PAM 3642
P14Δ*fosX* (*fosX*)	Δ*fosX* complemented with pPLP^{lmo1703}:*fosX*^{P14}	This study	PAM 4123
P14Δ*fosX* (P$^{\delta}$*fosX*)	Δ*fosX* complemented with pPLPδ:*fosX*^{P14}	This study	PAM 3645
P14Δ*fosX*Δ*hpt* (*fosX*)	Δ*fosX*, Δ*hpt* complemented with pPLP^{lmo1703}:*fosX*^{P14}	This study	PAM 4124
P14Δ*fosX* (*fosX*^{128stop})	P14 Δ*fosX* complemented with pPLPδ:*fosX*^{128stop}	This study	PAM 3698
P14Δ*fosX* (*fosX*^{88frameshift})	P14Δ*fosX* complemented with pPLPδ:*fosX*^{88frameshift}	This study	PAM 3696
P14 *prfA**	PrfA^{*G145S} derivative of P14	Our laboratory [38]	PAM 50
P14 *prfA**Δ*hpt*	In-frame *hpt* deletion mutant	Our laboratory [17,19]	PAM 377
P14 *prfA**Δ*fosX*	In-frame *fosX* deletion mutant	This study	PAM 3643
P14 *prfA**Δ*fosX*Δ*hpt*	In-frame double *hpt* and *fosX* deletion mutant	This study	PAM 3644
P14 *prfA**Δ*fosX* (*fosX*)	Δ*fosX* complemented with pPLP^{lmo1703}:*fosX*^{P14}	This study	PAM 4125
P14 *prfA**Δ*fosX*Δ*hpt* (*fosX*)	Δ*fosX*, Δ*hpt* complemented with pPLP^{lmo1703}:*fosX*^{P14}	This study	PAM 4126
Listeria innocua			
CLIP 11262	Wild-type	Institut Pasteur	PAM 3089
fosX::pLSV1	*fosX* knock-out mutant by plasmid insertion	This study	PAM 3914
fosX::pLSV-Rev	Plasmid-cured derivative from *L. innocua fosX::*pLSV	This study	PAM 3915
Escherichia coli			
DH5α	Cloning host strain		
**Plasmids**			
pMAD	Thermosensitive shuttle vector for allelic exchange	M. Debarbouille [77]	
pLSV1	Thermosensitive shuttle vector for allelic exchange	J. Kreft [79]	
pPL2	Integrative vector for stable gene complementation	M. Loessner [78]	
pMADΔ*fosX*	pMAD inserted with Δ*fosX* allele from P14	This study	
pLSV*fosX*^{Lin}	pLSV1 inserted with *fosX* from *L. innocua*	This study	PAM 3912
pPLP^{lmo1703}:*fosX*^{P14}	pPL2 inserted with *fosX* from P14 expressed from *lmo1703* promoter	This study	PAM 4128
pPLPδ:*fosX*^{P14}	pPL2 inserted with *fosX* from P14 expressed from Pδ promoter	This study	PAM 3648
pPLPδ:*fosX*^{128stop}	pPL2 inserted with *fosX* from strain 2007/1093 expressed from Pδ	This study	PAM 4141
pPLPδ:*fosX*^{88frameshift}	pPL2 inserted with *fosX* from strain 2007/0922 expressed from Pδ	This study	PAM 4142
