# Supplementary material for: Epistatic control of intrinsic resistance by virulence genes in Listeria
Source: PLoS Genet. 2018 Sep 4;14(9):e1007525. doi: 10.1371/journal.pgen.1007525 (PMC6122793; doi:10.1371/journal.pgen.1007525)
Supplement: S3 Table — Human isolates with wild-type fosfomycin susceptibility pattern (resistant in BHI and susceptible in BHI-Ads), including a selection of strains showing lowest MICs in the resistance range in BHI, were also sequenced as controls. Reference strains: P14, P14 prfA* and EGDe. Two other well-characterized L. monocytogenes strains (10403S and CLIP 80459) were also analyzed. (PDF) [file pgen.1007525.s007.pdf]

S3 Table. Analysis of PrfA-dependent phenotype and *fosX* genotype of *L. monocytogenes* clinical isolates constitutively susceptible to fosfomycin (BHI MIC ≤64 µg/ml). Human isolates with wild-type fosfomycin susceptibility pattern (resistant in BHI and susceptible in BHI-Ads), including a selection of strains showing lowest MICs in the resistance range in BHI, were also sequenced as controls. Reference strains: P14, P14 *prfA** and EGDe. In addition, two other well characterized *L. monocytogenes* strain (10403S and CLIP 80459) were analyzed.

Strain [a]	Other designations [b]	Fosfomycin phenotype [c]	MIC (µg/ml) [d]		Sugar-phosphate utilization [e]	FosX sequence type [g]	*fosX* mutation
			BHI	BHI-Ads			
P14 (PAM 14)		wt	≥1024	24	–	P14	na [h]
P14 *prfA** (PAM 50)		sfs	12	1.4	+	P14	None
EGDe (PAM 169)		wt	128/192	2	–	EGDe	na
10403S (PAM 170)		wt	768	2	–	EGDe	na
CLIP 80459 (PAM 3056)		wt	≥1024	24	–	P14	na
PAM 3443	CLIP 01786	sfs	24	1	–	EGDe	L128 → stop codon
PAM 3446	CLIP 01801	sfs	24	2	–	EGDe	L128 → stop codon
PAM 3393	CLIP 80731	sfs	24	1	(+) [f]	P14	None
PAM 3420	CLIP 2007/00092	sfs	32	3	–	P14	S88 → frame-shift mutation
PAM 3358	CLIP 2008/00244	sfs	48	4	–	EGDe	L128 → stop codon
PAM 3349	CLIP 2007/01366	sfs	48	8	–	EGDe	L128 → stop codon
PAM 3412	CLIP 2007/01068	sfs	64	4	–	EGDe	L128 → stop codon
PAM 3415	CLIP 2007/01093	sfs	64	2	(+)	EGDe	L128 → stop codon, E24K
PAM 3355	CLIP 2007/00702	sfs	64	1	–	EGDe	L128→ stop codon
PAM 3352	CLIP 2007/00727	wt	256	4	–	EGDe	None
PAM 3389	CLIP 2007/00855	wt	384	12	–	EGDe	None
PAM 3391	CLIP 2008/00370	wt	512	8	–	EGDe	None
PAM 3390	CLIP 2007/00877	wt	192	8	–	EGDe	None
PAM 3340	CLIP 20969	wt	768	4	–	P14	None
PAM 3356	CLIP 2007/00823	wt	≥1024	12	–	P14	None
PAM 3382	CLIP 2008/00117	wt	192	12	–	EGDe	None
PAM 3353	CLIP 2007/00511	wt	256	4	–	EGDe	None

[a] Internal isolate collection number of JV-B laboratory.
[b] Collection of *Listeria* of Institut Pasteur CLIP number (National Reference Center and WHO Collaborating Center for *Listeria*, Institut Pasteur, Paris).
[c] wt = wild-type susceptibility pattern (resistant in BHI, susceptible in BHI-Ads), sfs = spontaneously susceptible (in BHI); susceptibility breakpoint ≤64 µg/ml (see text).
[d] Determined using E-test in BHI and charcoal-supplemented BHI (BHI-Ads). MIC values from at least two determinations.
[e] Determined in phenol-red medium after 24 h incubation at 37 ºC. All strains were positive using charcoal-supplemented phenol red medium.
[f] Indicates intermediate ("slow positive") sugar acidification reaction (orange color instead of bright yellow for a positive; negative, red).
[g] The wild-type FosX sequence of strain EGDe differs at 10 positions from that of strain P14 (A20T, Q23R, N24E, K37Q, R65Q, Q73R, T83I, A88S, M93I, Q101E). The P14 and EGDe sequences are the prototypes of the lineage I and lineage II FosX sequence types; see S1 Table.
[h] na, not applicable.
