# Supplementary material for: Epistatic control of intrinsic resistance by virulence genes in Listeria
Source: PLoS Genet. 2018 Sep 4;14(9):e1007525. doi: 10.1371/journal.pgen.1007525 (PMC6122793; doi:10.1371/journal.pgen.1007525)
Supplement: S4 Table — Relevant restriction sites are underlined. (PDF) [file pgen.1007525.s008.pdf]

S4 Table. Main nucleotides used in this study. Relevant restriction sites are underlined.

Primer	Sequence 5´ → 3´	Use
pMAD1	AGTCGAACACTCCATCAGAC	pMAD checking
pMAD2	TGATCGAAGTTAGGCTGGTA	
PL95	ACATAATCAGTCCAAAGTAGATGC	Checking insertion of pPL2 into listerial chromosome
NC16	GTCAAAACATACGCTCTTATC	
fosXDM-1 NcoI	GCCCATGGAACATCCTTCTGGTC	Δ*fosX* deletion
fosXDM-2 SalI	GCGTCGACAGGTATCACGAATAG	
fosXDM-3 SalI	GCGTCGACTAATCCTGAAATCATGT	
fosXDM-4 BamHI	GCGGATCCAGTGGTATCGT	
fosXDM-5	CTATCTCAAGTGGTACGGAACG	Checking for Δ*fosX* deletion
fosXDM-6	TGGTAGGCAGTGGTCAAGT	
Plmo1703 F2 SalI	GCGGTCGACCTGTTCGATGGTTCGTTCTGTC	Construction of pPL2P^{Lmo1702} *fosX*
Plmo1703-NdeI R	GGGAATTCCATATGTATTTTCCTCCATCATTCC	
fosX-ATG-NdeI F	GGGAATTCCATATGATTTCAGGATTAAGCCAT	
fosX-Comp R SacI	CCCGAGCTCGGCGCGAATTCATTGTCG	
FosXFBamHI-pPL2Pdelta	CGGGATCCCTGTAGTACTCTT	Construction of pPL2P$_{\delta}$*fosX*
FosXRSpeI-pPL2Pdelta	GGACTAGTTCATTCGTTTCATC	
Li-fosX-Int-F1	CGGGATCCCGCAGGATTAAGTCATATCACT	Construction of *L. innocua fosX::*pLSV1
Li-fosX-Int-R1-EcoRI	CCGGAATTCCGGATGTCCCGGCGTGTAATTC	
Li-fosX-out-F	TGGCAGTGGGTTCAGGTGG	Checking insertion of *fosX::*pLSV1 into *L. innocua* chromosome
Li-fosX-out-R	CACGATGGCGGACCATTATGG	
qPCR-actA F	AAGAAATTGATCGCCTAGCTGATT	qPCR of *actA*
qPCR-actA R	GTAAAAAACCCGCATTTCTTGAGT	
qPCR-actA P	FAM-TTTCCTGTTCCTCTATCTCT-MGB-BHQ1	
qPCR-ldh F	ATGCTCGTAACGTCCATGGTT	

Primer	Sequence 5′ → 3′	Use
qPCR-ldh R	GCTCCATGCTGGGAATTCTG	qPCR of *ldh*
qPCR-ldh P	FAM-CATCCTTGGCGAACACGGCGA-TAMRA	
qPCR-rpoB F	TGGTTCTTAGATGAAGGGCTACGT	
qPCR-rpoB R	ACCCGCAAAATCCTCAATTG	qPCR of *rpoB*
qPCR-rpoB P	FAM-AATATCGCGGAACATCT-MGB-NFQ	
qPCR fosX F	AGCTCTCGGTGTGGAAATGA	qPCR of *fosX*
qPCR fosX R	CGTTCTTCTAATGTACCAGCGT	

Restriction enzymes recognition sites are underlined
